# Emergence of Double- and Triple-Gene Reassortant G1P[8] Rotaviruses Possessing a DS-1-Like Backbone after Rotavirus Vaccine Introduction in Malawi

**DOI:** 10.1128/JVI.01246-17

**Published:** 2018-01-17

**Authors:** Khuzwayo C. Jere, Chrispin Chaguza, Naor Bar-Zeev, Jenna Lowe, Chikondi Peno, Benjamin Kumwenda, Osamu Nakagomi, Jacqueline E. Tate, Umesh D. Parashar, Robert S. Heyderman, Neil French, Nigel A. Cunliffe, Miren Iturriza-Gomara

**Affiliations:** a Institute of Infection and Global Health, University of Liverpool, Liverpool, United Kingdom; b Malawi-Liverpool-Wellcome Trust Clinical Research Programme/Department of Medical Laboratory Sciences, College of Medicine, University of Malawi, Blantyre, Malawi; c Centre for Global Vaccine Research, Institute of Infection and Global Health, University of Liverpool, Liverpool, United Kingdom; d Graduate School of Biomedical Sciences, Nagasaki University, Nagasaki, Japan; e Epidemiology Branch, Division of Viral Diseases, National Center for Immunization and Respiratory Diseases, Centers for Disease Control and Prevention, Atlanta, Georgia, USA; f Division of Infection and Immunity, University College London, London, United Kingdom; g NIHR Health Protection Research Unit in Gastrointestinal Infections, University of Liverpool, Liverpool, United Kingdom; Loyola University Medical Center

**Keywords:** rotavirus, phylodynamics, genome reassortment, lineage turnover, whole-genome sequencing, Malawi

## Abstract

To combat the high burden of rotavirus gastroenteritis, multiple African countries have introduced rotavirus vaccines into their childhood immunization programs. Malawi incorporated a G1P[8] rotavirus vaccine (Rotarix) into its immunization schedule in 2012. Utilizing a surveillance platform of hospitalized rotavirus gastroenteritis cases, we examined the phylodynamics of G1P[8] rotavirus strains that circulated in Malawi before (1998 to 2012) and after (2013 to 2014) vaccine introduction. Analysis of whole genomes obtained through next-generation sequencing revealed that all randomly selected prevaccine G1P[8] strains sequenced (*n* = 32) possessed a Wa-like genetic constellation, whereas postvaccine G1P[8] strains (*n* = 18) had a DS-1-like constellation. Phylodynamic analyses indicated that postvaccine G1P[8] strains emerged through reassortment events between human Wa- and DS-1-like rotaviruses that circulated in Malawi from the 1990s and hence were classified as atypical DS-1-like reassortants. The time to the most recent common ancestor for G1P[8] strains was from 1981 to 1994; their evolutionary rates ranged from 9.7 × 10^−4^ to 4.1 × 10^−3^ nucleotide substitutions/site/year. Three distinct G1P[8] lineages chronologically replaced each other between 1998 and 2014. Genetic drift was the likely driver for lineage turnover in 2005, whereas replacement in 2013 was due to reassortment. Amino acid substitution within the outer glycoprotein VP7 of G1P[8] strains had no impact on the structural conformation of the antigenic regions, suggesting that it is unlikely that they would affect recognition by vaccine-induced neutralizing antibodies. While the emergence of DS-1-like G1P[8] rotavirus reassortants in Malawi was therefore likely due to natural genotype variation, vaccine effectiveness against such strains needs careful evaluation.

**IMPORTANCE** The error-prone RNA-dependent RNA polymerase and the segmented RNA genome predispose rotaviruses to genetic mutation and genome reassortment, respectively. These evolutionary mechanisms generate novel strains and have the potential to lead to the emergence of vaccine escape mutants. While multiple African countries have introduced a rotavirus vaccine, there are few data describing the evolution of rotaviruses that circulated before and after vaccine introduction. We report the emergence of atypical DS-1-like G1P[8] strains during the postvaccine era in Malawi. Three distinct G1P[8] lineages circulated chronologically from 1998 to 2014; mutation and reassortment drove lineage turnover in 2005 and 2013, respectively. Amino acid substitutions within the outer capsid VP7 glycoprotein did not affect the structural conformation of mapped antigenic sites, suggesting a limited effect on the recognition of G1-specific vaccine-derived antibodies. The genes that constitute the remaining genetic backbone may play important roles in immune evasion, and vaccine effectiveness against such atypical strains needs careful evaluation.

## INTRODUCTION

Diarrhea is a leading cause of mortality in children under the age of 5 years globally (1, 2). The majority of hospitalizations and deaths of infants due to severe dehydrating diarrhea are caused by group A rotaviruses (RVAs) (3). The World Health Organization (WHO) recommended the universal introduction of rotavirus vaccines in 2009, particularly in countries where the diarrhea mortality rate is high (4). A global decline from 528,000 to 215,000 rotavirus-associated deaths per year among children <5 years of age between 2000 and 2013 has been reported, and live-attenuated oral rotavirus vaccines (Rotarix [RV1] and RotaTeq [RV5]) have now been incorporated into the national immunization programs of over 60 countries worldwide (5).

RVAs are members of the *Reoviridae* virus family. They are enveloped icosahedric viruses that contain a triple-layered capsid encasing 11 genome segments of double-stranded RNA (dsRNA). The rotavirus genome encodes six structural proteins (VP1 to VP4, VP6, and VP7) and five to six nonstructural proteins (NSP1 to NSP5/NSP6) (6). Nucleotide homology cutoff values for the open reading frame (ORF) for each genome segment are used to classify rotavirus strains on the basis of the whole-genome composition (7, 8). To date, 35 G (VP7), 50 P (VP4), 26 I (VP6), 21 R (VP1), 19 C (VP2), 19 M (VP3), 30 A (NSP1), 20 N (NSP2), 21 T (NSP3), 26 E (NSP4), and 21 H (NSP5) genotypes have been described (8–12). (http://rega.kuleuven.be/cev/viralmetagenomics/virus-classification).

Genotypes G1 to G4, G9, and G12 in association with genotype P[4], P[6], or P[8] are the predominant genotypes associated with human rotavirus infection worldwide (6, 13–15). Although several G and P genotype combinations have been detected among human rotaviruses, the genotypes for the other nine genes are limited to predominantly genotype 1 (I1-R1-C1-M1-A1-N1-T1-E1-H1; Wa-like) and genotype 2 (I2-R2-C2-M2-A2-N2-T2-E2-H2; DS-1-like) (16). For instance, typically, G1P[8], G3P[8], G4P[8], G9P[8], and G12P[8] RVAs have a Wa-like genotype constellation, whereas G2P[4] and G8P[4] or G8P[6] strains usually possess a DS-1-like constellation (16–18). The segmented RNA genome of rotaviruses and their error-prone RNA-dependent RNA polymerase, which lacks proofreading activity (6), allow various evolutionary mechanisms, including genetic mutation, recombination, and reassortment. This leads to the emergence of distinct lineages within individual genotypes or reassortant viruses containing segments from different progenitor strains (6, 19, 20).

Novel double-reassortant DS-1-like G1P[8] rotaviruses have recently emerged in Southeast Asia. These atypical G1 strains were initially detected in outbreaks of gastroenteritis among Japanese children (21–23), followed by reports from Thailand (24, 25) and then Vietnam (26). To date, there is no evidence that these atypical G1 strains are widespread. Rotavirus strain surveillance conducted in Blantyre, Malawi, since 1997, and the introduction of the monovalent, Wa-like G1P[8] rotavirus vaccine (Rotarix or RV1) into Malawi's childhood immunization program in 2012 allow the study of the evolution of G1P[8] strains before and after vaccine introduction. We have, for the first time, detected DS-1-like G1P[8] rotavirus strains in Africa, which became predominant following vaccine introduction. The evolutionary forces that were associated with the emergence of the atypical G1P[8] rotavirus strains were also determined.

## RESULTS

### Emergence of reassortant DS-1-like G1P[8] rotavirus strains.

**FIG 1:**
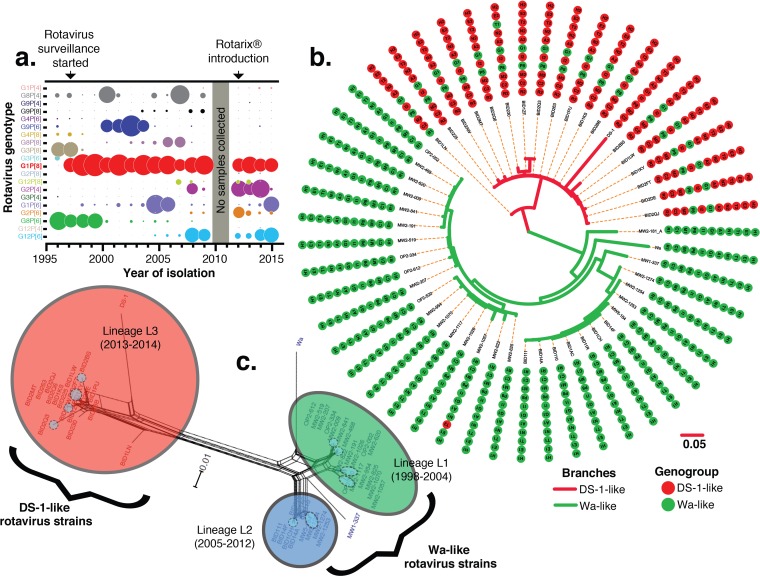
Rotavirus strains, genetic constellations, and phylogenetic networks of G1P[8] rotaviruses detected in Malawian children at QECH from 1997 to 2015. (a) Schematic representation of the proportions of all genotypes detected in rotavirus-positive stool samples. The size of the circle is directly proportional to the frequency of detection of G and P genotypes. There were no rotavirus surveillance activities in 2010; hence, samples were not collected at this time. (b) Bayesian maximum clade credibility tree for concatenated whole-rotavirus-genome sequences illustrating the genetic constellation and reassortment patterns for 32 pre- and 18 postvaccine G1P[8] strains from Malawi as well as the prototype Wa and DS-1 strains for comparison. Genotype and genogroup assignments for each segment were based on nucleotide sequence identities, assigned by using RotaC. Genome segments that were assigned the same genotype are shown with the same color and genotype numbers. (C) Phylogenetic network of complete concatenated whole-genome sequences of G1P[8] rotavirus strains detected in Malawi from 1998 to 2014. Branches are drawn to scale, and splits in the network indicate reassortments. Network clusters are color-coded and named in accordance with their phylogenic lineages (L1 to L3) that correlated with the time of strain isolation before or after rotavirus vaccine introduction. Clusters L1 (green) and L2 (blue) contain prevaccine G1P[8] strains, whereas L3 (red) contains postvaccine strains. Network subclusters within each main cluster are shaded in blue (L1), red (L2), or orange (L3).

All prevaccine (33.4%; 1,634/4,888) and postvaccine (22.6%; 477/2,109) rotavirus-positive stool samples collected from children presenting with acute severe diarrhea at Queen Elizabeth Central Hospital (QECH) in Blantyre, Malawi, were genotyped as part of ongoing rotavirus surveillance (27–30). Multiple strains were characterized, and G1P[8] rotaviruses were consistently predominant strains that were detected each year before (39.4%; 554/1406) and after (31.4%; 95/303) vaccine introduction (Fig. 1a; see also Fig. S1 in the supplemental material). Whole-genome sequences of 32 prevaccine G1P[8] strains (collected between 1998 and 2012) and 18 postvaccine G1P[8] strains (collected from 2013 to 2014) were successfully generated (see Table S1 in the supplemental material for the yearly distribution). Interruption of surveillance in 2010 meant that no G1P[8] strains were available between 2010 and 2011. Among the prevaccine G1P[8] strains, 31 had the G1-P[8]-I1-R1-C1-M1-A1-N1-T1-E1-H1 genotype constellation and hence were designated Wa-like G1P[8] strains, whereas 1 had the G1-P[8]-I1-R1-C1-M1-A1-N1-T2-E1-H1 genotype constellation and hence was designated a monoreassortant Wa-like G1P[8] strain. In contrast, 16/18 of the postvaccine G1P[8] strains had a DS-1-like genotype constellation (G1-P[8]-I2-R2-C2-M2-A2-N2-T2-E2-H2) and hence were designated atypical double-reassortant DS-1-like G1P[8] strains. The remaining two postvaccine G1P[8] strains (BID1LN and BID230) had Wa-like VP1 (R1) and Wa-like NSP3 (T1) genes, respectively, and hence were designated atypical triple-reassortant DS-1-like G1P[8] strains (Fig. 1b and Table S1).

### Atypical postvaccine G1P[8] strains emerged through genome reassortment between Wa-like and DS-1-like human rotaviruses.

While the concatenated sequences of all 11 genome segments of pre- and postvaccine G1P[8] rotaviruses clustered with prototype Wa- and DS-1-like strains, respectively (Fig. 1b), phylogenetic networks constructed by using characterized concatenated whole-genome sequences of G1P[8] strains revealed frequent reassortment events between strains from the same or different network clusters (Fig. 1c). The Malawi G1P[8] strains were distributed into three main phylogenetic network clusters (L1, L2, and L3), which also contained multiple subclusters (shaded within main clusters in Fig. 1c). The L1 phylogenetic cluster contained strains that circulated from 1998 to 2004, and strains detected from 2005 to 2012 were grouped into the L2 cluster, while the L3 cluster consisted only of postvaccine G1P[8] strains that circulated from 2013 to at least 2014 in Blantyre.

The phylogenetic relationship of Malawi G1P[8] strains was inferred by using the maximum likelihood method, for which complete nucleotide sequences of RVAs available in GenBank and sequences of Wa-like (VP4 and VP7 only) and DS-1-like (the other nine genes) strains from Malawi were used. The DS-1-like genes of the postvaccine G1P[8] reassortants clustered closely with those of G2 strains that circulated simultaneously in Malawi (see Fig. S2 in the supplemental material) and not with DS-1-like G1P[8] strains identified in Southeast Asia and Japan. Sequences within each cluster were 95 to 99.8% similar (calculated by using nucleotide identity matrices [data not shown]).

All 11 genome segments of the G1P[8] strains were undergoing purifying selection, thus potentially resulting in stabilizing selection following the purging of deleterious variants arising during error-prone rotavirus replication due to its RNA genome (Table 1). The genetic algorithm for recombination detection (GARD) (31) and single-breakpoint recombination (SBP) (32) did not identify any recombination events within each genome segment of the study strains (Table 1). Thus, the changes in the genetic constellation of postvaccine G1P[8] strains were likely generated through the exchange of a whole gene segment (reassortment) between circulating Wa-like and DS-1-like human rotaviruses.

**TABLE 1:** Evolutionary selective forces and recombination in all 11 proteins of the Malawian G1P[8] rotavirus strains^*a*^

Protein	*dN/dS* by SLAC	ω (β/α) by FUBAR
VP1	0.0502	2.22
VP2	0.0538	2.67
VP3	0.0965	2.47
VP4	0.1052	3.15
VP6	0.0310	4.66
VP7	0.2033	6.01
NSP1	0.2239	4.21
NSP2	0.0920	5.62
NSP3	0.0870	5.44
NSP4	0.1097	7.10
NSP5	0.1097	7.47

a Shown are posterior distributions of synonymous (α) and nonsynonymous (β) substitution rates over sites as well as mean posterior probabilities for a ω (β/α) value of <1 at a site. The consensus selective force in all cases was purifying selection, and there was no recombination determined by GARD or SBP.

### G1P[8] strains that circulated in Malawi between 1998 and 2014 exhibit distinct replacement dynamic patterns.

**FIG 2:**
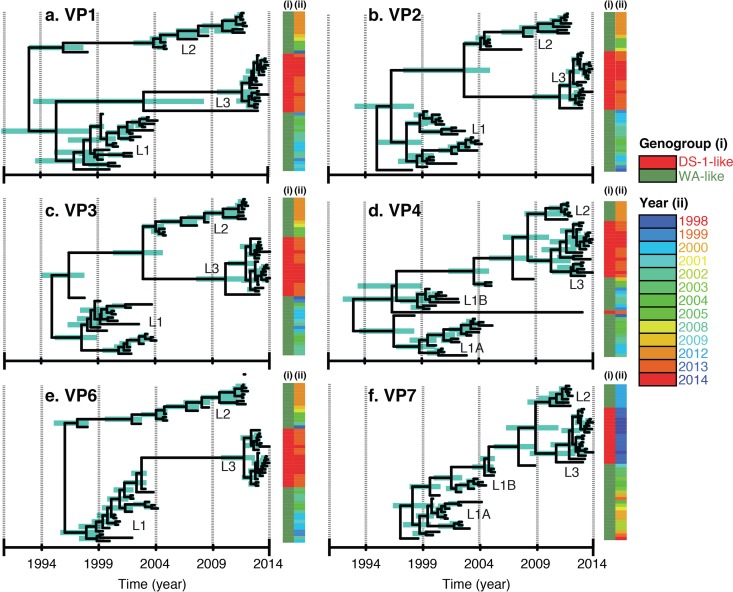
Bayesian maximum clade credibility (MCC) time tree based on complete nucleotide sequences illustrating lineage replacement within the genome segments encoding structural proteins of the G1P[8] strains that circulated in Malawi from 1998 to 2014. With the exception of VP4 and VP7 genes that had L2 and L3 genes sharing close ancestry, the rest had three distinct G1P[8] lineages. L1, L2, and L3 represent lineages 1, 2, and 3, respectively.

Bayesian inferences of time-measured trees were individually constructed for each of the 11 genome segments of the G1P[8] strains to further determine their evolutionary dynamics. As illustrated by phylogenetic networks, prior to the emergence of the reassortant L3 lineage, both structural and nonstructural genes segregated into at least 2 distinct lineages with a common ancestor in the mid-1990s or before (Fig. 2; see also Fig. S3 in the supplemental material). For NSP1, NSP2, NSP3, VP1, VP2, VP3, and VP6, a single lineage (L1) was predominant until the mid-2000s (2003 to 2005), and following its disappearance, strains forming a second lineage, L2, circulated until the emergence of the DS-1-like G1P[8] reassortant strains. For the VP4, VP7, and NSP4 genes, two clusters cocirculated until 2004, and replacement with strains in L2 did not occur until later, between 2010 and 2012. In 2013, VP7- and VP4-encoding genes of the emergent reassortant strains also formed a third cluster with a likely common ancestor with strains in L2. Both maximum clade credibility (MCC) coalescent framework and maximum likelihood phylogenetic approaches showed that G1P[8] strains acquired DS-1-like genome segments (genotype 2) in the nine non-VP4 and non-VP7 genes in 2013, outcompeting typical Wa-like L2 variants (genotype 1) during the postvaccine era (Fig. 2 and Fig. S2 to S4). These analyses also showed that the post-2013 Malawi DS-1-like G1P[8] reassortant strains clustered with or were derived from Wa-like G1P[8] strains and DS-1-like G2 strains that cocirculated in Malawi and hence emerged through reassortment among local strains. The genes of Malawi DS-1-like G1P[8] strains clustered away from those of DS-1-like reassortants that emerged recently in southeast Asian countries and Japan, indicating that they were likely not imported.

**FIG 3:**
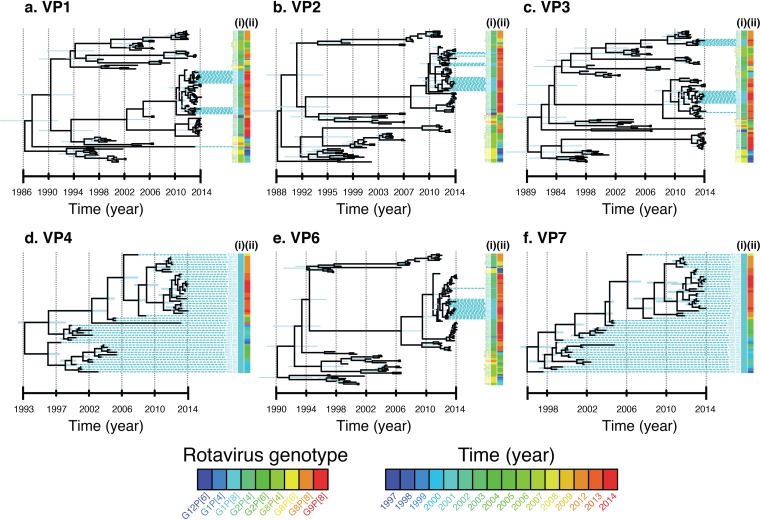
Bayesian MCC time tree based on complete nucleotide sequences of the structural proteins for G1P[8] strains from Malawi. Only DS-1-like genome segments for typical DS-1-like strains that were assigned G2P[4], G2P[6], G8P[4], G8P[6], and G12P[6] outer capsid genotypes from Malawi were included to calculate evolutionary dynamics for VP1- to VP4-, VP6-, and VP7-encoding genome segments for the atypical DS-1-like G1P[8] strains (L3 cluster).

**FIG 4:**
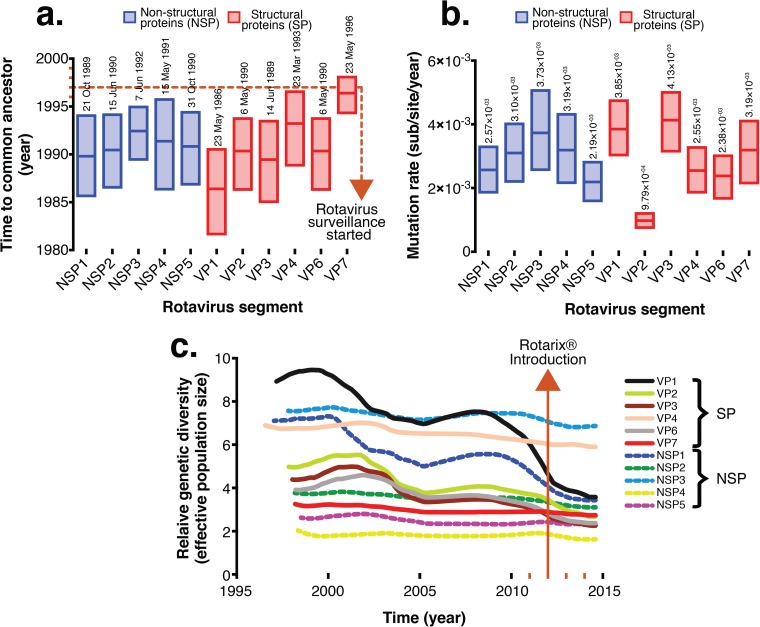
Time to the most recent common ancestor (tMRCA), evolutionary rates for each genome segment of Malawian G1P[8] strains, and comparative population dynamics of G1P[8] rotavirus strains circulating in Malawi, 1998 to 2014. (a) Evolutionary rates and tMRCAs for each genome segment of the atypical Malawian G1P[8] strains shown together with their 95% highest posterior density (HPD) intervals. (b) Absolute values for the means and ranges of evolutionary rates and tMRCA at 95% HPD intervals. (c) Phylogenies and relative genetic diversities estimated by using the Gaussian Markov random field (GMRF) model represent Bayesian Skygrid plots for VP1- to VP4-, VP6-, and NSP1- to NSP5-encoding genome segments. Lines in the GMRF plot represent the mean relative genetic diversity through time.

Since the postvaccine Malawi G1P[8] strains contained a DS-1-like genetic backbone, only sequences for the Wa-like genome segments for G1P[8] strains generated in this study were used for phylodynamic analysis of VP4 and VP7 genes, whereas cognate genes of DS-1-like strains that were assigned various G and P types (Table S1), which also circulated in Malawi from 1997 to 2014, were used to estimate evolutionary dynamics for non-VP4 and non-VP7 genes for postvaccine G1P[8] strains (Fig. 3 and Fig. S4). The calculated mean times per gene to the most recent common ancestor (tMRCAs) ranged from the years 1986 to 1996 (Fig. 4a). When only non-VP4 and non-VP7 genes (NSP1 to NSP4, VP1 to VP3, and VP6) for reassortant DS-1-like G1P[8] and cognate genes of DS-1-like strains collected from Malawi between 1997 and 2014 were used to infer Bayesian time-measured trees, the tMRCA for the atypical G1P[8] strains (L3 cluster) was estimated to range from 2009 to 2011, which was similar to predominantly DS-1-like G2 strains that were detected after vaccine introduction (Fig. 3 and Fig. S4). Marginal differences between the mean evolutionary rates for each genome segment were observed, which ranged from 9.7 × 10^−4^ to 4.1 × 10^−3^ nucleotide substitutions per site per year (Fig. 4b). VP2 had the lowest mutation rates (9.7 × 10^−4^ nucleotide substitutions per site per year; 95% highest posterior density [HPD] interval, 7.4 × 10^−4^ to 1.2 × 10^−3^ substitutions per site per year), whereas VP3 had the highest mutation rates (4.1 × 10^−3^ substitutions per site per year; HPD, 3.1 × 10^−3^ to 5 × 10^−3^ substitutions per site per year) (Fig. 3 and 4b and Fig. S4).

We then utilized a Gaussian Markov random field (GMRF) tree prior to investigate whether Rotarix introduction had an impact on the relative population size of the circulating G1P[8] strain. There was no evidence to suggest that vaccine introduction affected either G1P[8] genetic diversity or population size for the VP4, VP7, NSP2, NSP3, and NSP5 genes, as the peaks and troughs of their Skygrid plots exhibited similar stable profiles just before (2005 to 2012) and after (2013 to 2014) vaccine use. In contrast, genes encoding VP1 to VP3, VP6, and NSP1 had relatively stable profiles and also small effective population sizes during the postvaccine era compared to those before vaccine introduction, which could be natural, as similar downward trends were already occurring before vaccine introduction (Fig. 4c).

### Mutations within VP7 antigenic regions did not affect the structural conformation of neutralizing epitopes essential for antigenic recognition by neutralizing antibodies.

**FIG 5:**
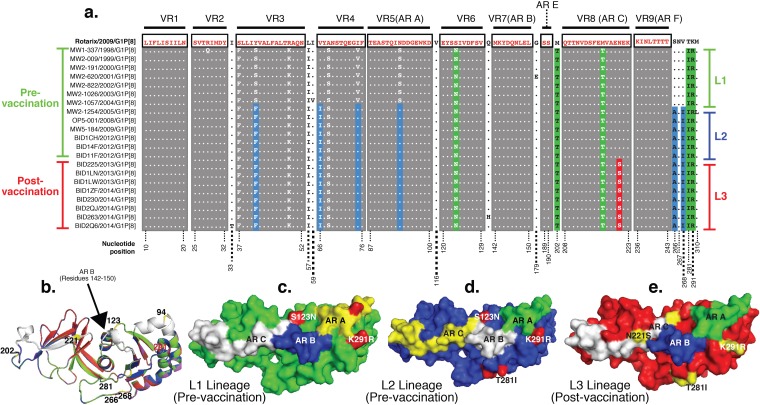
Amino acid substitutions and structural conformation of the outer capsid glycoprotein of Malawian G1P[8] strains. (a) Complete VP7 sequences of representative pre- and postvaccine G1P[8] strains aligned to that of RV1 exhibiting amino acid substitutions that occurred within the variable regions (VR) and mapped antigenic regions (AR) over time. Lineage-defining amino acid amino acid substitutions are highlighted in green, blue, and yellow for the L1, L2, and L3 lineages, respectively. Pre- and postvaccine strains are shown with vertical green and red bars on the right, respectively. Strains belonging to the L1, L2, and L3 phylogenetic clusters are shown with green, blue, and red bars, respectively, on the right. (b) Perfect alignment of superimposed VP7 structures exhibiting few differences between RV1 and L1 to L3 strains. Antigenic regions A, B, and C are shown in white. L1 to L3 and RV1 strains are shown in yellow, green, blue, and red, respectively. (c to e) Surface visualization of VP7 from outside the virion on the 3-fold axis displaying amino acid differences when structures for L1 (c), L2 (d), and L3 (e) G1P[8] strains were superimposed on the structure of the outer capsid glycoprotein of RV1. Numbers correspond to the positions where mutations occurred.

Mapped amino acid motifs that constitute neutralizing epitopes on the outer capsid glycoprotein were compared between Rotarix and Malawian G1P[8] strains. In total, 15 lineage-defining amino acid substitutions were identified across the entire VP7 sequence, with only 5 of these being located at mapped antigenic regions 7-1a (S123N and K291R) and 7-2 (AR C [M202T, M212T, and N221S]) (Fig. 5a). A single amino acid substitution, N221S, located at one of these antigenic regions, differentiated L3 from L2 cluster strains (Fig. 5).

The VP7 structures of L1 to L3 strains and that of the Rotarix strain aligned perfectly when superimposed (Fig. 5b), implying a conserved conformation consistent with the conservation in chemical properties of the replacing amino acids. The replacing amino acids did not appear to impact the structural conformation of the antigenic regions of the glycocapsid protein of the G1P[8] strains circulating before and after vaccine introduction (Fig. 5c to e).

## DISCUSSION

Malawi was one of the first African countries to introduce a rotavirus vaccine into its infant immunization schedule in October 2012. By 2015, vaccine coverage had exceeded 95% (30). We enhanced rotavirus surveillance activities in the postvaccine period in Malawi primarily to assess the impact of vaccine introduction on the burden of rotavirus disease (27, 30). The availability of a collection of rotavirus strains isolated before (1997 to 2012) (28) and after Rotarix introduction offered a rare opportunity to assess the early impact of vaccine introduction on the genetic diversity of circulating strains and their evolutionary patterns over time. In the present study, phylogenetic analysis and evolutionary history were inferred for G1P[8] rotaviruses, the most prevalent rotavirus strains globally (6). In Malawi, G1P[8] strains were the only rotaviruses consistently detected year to year, from 1998 to 2014, hence enabling a systematic inference of evolutionary patterns over time. Furthermore, G1P[8] strains are homologous, with respect to VP7 and VP4 genotypes, to the Rotarix vaccine that is in use in Malawi (33), hence permitting homologous genomic comparison.

While the prevaccine G1P[8] strains had a typical Wa-like genetic constellation (8, 16, 34, 35), the backbone of the G1P[8] strains detected after vaccine introduction possessed a DS-1-like genotype constellation, which is associated most frequently with G2P[4] strains, occasionally with G9 and G12 strains, and with G8P[4] or G8P[6] strains in Africa (8, 17, 18, 36). The detection of atypical reassortant DS-1-like G1P[8] strains had not been documented during 15 years of prevaccine rotavirus strain surveillance in Malawi or elsewhere in Africa. Similar atypical reassortant DS-1-like G1P[8] strains emerged recently in Southeast Asian countries where rotavirus vaccine use is limited (21–26). Atypical DS-1-like G1P[8] strains from Malawi and Asia exhibited distinct phylogenetic clustering patterns in all 11 genome segments, indicating that these atypical DS-1-like G1P[8] strains most likely emerged independently in Malawi and Asia and did not arise through importation. In addition, (i) tight clustering between the G and P genes of the atypical Malawian G1P[8] strains with those of other G1P[8] Wa-like strains, (ii) monophyletic clustering between non-G and non-P genes of atypical Malawian G1P[8] strains and those of other Malawian DS-1-like strains, and (iii) evidence of frequent reassortment events among DS-1- and Wa-like strains circulating in Malawi during the study period as revealed by phylogenetic networks suggest that the atypical Malawian G1P[8] strains emerged locally through genome reassortment among cocirculating Wa- and DS-1-like strains. This is further supported by the detection of a high prevalence of G2 strains in Malawi from 2012 (27, 30), thus providing the required circulating strains to allow the emergence of Wa/DS-1-like reassortant strains.

The Malawian G1 mutation rate falls in the same range as those of rotavirus G9 and G12 strains (37–41). Zeller et al. (35) recently analyzed the phylodynamics of typical Wa-like G1P[8] strains that circulated before and after vaccine introduction in Belgium and Australia. Unlike the Malawian G1P[8] strains, all G1P[8] strains from Belgium and Australia had a Wa-like genetic constellation, and their tMRCA ranged from 1846 to 1945, whereas their evolutionary rates, which ranged from 6.05 × 10^−4^ to 1.01 × 10^−3^ nucleotide substitutions per site per year, were similar to those for Malawian G1P[8] strains. These rates are relatively lower than the known rates of RNA viruses (42), possibly due to the double-stranded nature of the rotavirus genome.

The emergence of the atypical reassortant DS-1-like G1P[8] strains in Malawi coincided with the programmatic rollout of Rotarix. Our data suggest that the atypical Malawian DS-1-like G1P[8] strains were derived from a combination of reassortment and drift of rotaviruses that were circulating locally since the 1990s. We found that at least three G1P[8] lineages were circulating in Malawi from 1998 to 2014. The diversity of the circulating G1P[8] variants exhibited periodic lineage replacements, similarly to influenza virus (43), dengue virus (44), and other enteric viruses such as noroviruses (45), where lineage replacement also appears to be an important evolutionary mechanism in response to herd immunity (35, 46, 47). Lineage diversity and replacement coincided temporally for blocks of genes and can be explained by drift and reassortment events occurring hand in hand.

Although the detection of DS-1-like G1P[8] strains coincided with the widespread use of a G1P[8] Rotarix rotavirus vaccine in Malawi in 2013, it is difficult to ascertain the role that the vaccine had in the emergence of these atypical strains considering the short postvaccine period. The data from phylodynamic analyses suggested that these strains were derived from those strains circulating in the human population well before vaccine introduction. It was difficult to determine the effect of vaccine introduction on the effective population size of the circulating G1P[8] strains, as only postvaccine G1 strains from 2013 and 2014 were analyzed, a time which was too early to detect genetic variations in the virus population size. The frequent detection of DS-1-like G2 strains just before Rotarix introduction and during the postvaccine era in Malawi (27, 30) may indicate a natural surge of DS-1-like rotaviruses during this period, similarly to G2 cyclic seasonal epidemic patterns that have been observed in many countries, including Africa (48, 49). However, the predominance of reassortant DS-1-like G1P[8] strains after vaccine introduction could suggest that there is positive selection for atypical G1P[8] strains and that such selection may not be driven exclusively by the VP7 and VP4 specificities. The DS-1-like genotype constellation was, however, found in rotavirus diarrhea cases regardless of the vaccination status of the children (48% were vaccinated [data not shown]). The relatively fewer rotavirus G1P[8] genomes that were analyzed are a potential limitation of the present study; as such, sequencing of additional G1P[8] strains that circulated in Malawi post-2014 is under way to determine whether emerging DS-1-like G1P[8] strains remain in circulation.

It was shown previously that mutations along the three main mapped neutralizing epitopes of VP7 can generate vaccine escape mutants (50). For instance, amino acid substitutions at positions 94, 97, 147, and 291 significantly affect the antigenic recognition of human G1 strains (50). When VP7 sequences of postvaccine G1P[8] strains (L3) were compared to that of Rotarix, only the K291R (7-1a) substitution occurred within the sites associated with antibody escape mutants, and this substitution was already present among the strains circulating before vaccine introduction since 1998 and hence was not selected due to potential vaccine pressure. Furthermore, both lysine (K) and arginine (R) are positively charged polar proteins; hence, this substitution is unlikely to produce significant changes in the biochemical properties of VP7. The only substitution present among the postvaccine G1 strains was N221S. As both asparagine (N) and serine (S) are small noncharged residues, this substitution does not appear to have a significant impact on the overall conformation and structure of the protein surface. However, the loss of an asparagine residue may have resulted in the loss of a potential glycosylation site, which could affect VP7 antigenic determinants (51). This change does not appear to be a universal glycosylation position, as serine also occurs naturally at position 221 for some non-G1 strains like S2 (G2), RV-5 (G3), and ST5 (G4) strains (52). While this change is outside the currently proven glycosylation sites (positions 69 to 71, 238 to 240, and 318 to 320), the N221S change occurred within neutralizing epitope C (antigenic region C) of VP7 (52). In order to exclude the potential for this amino acid substitution, further functional studies may be warranted, bearing in mind that changes in immunogenicity and neutralization patterns have been attributed to different glycosylation patterns using mutated laboratory strains (51, 53).

The only hydrophobic-to-hydrophilic change, which would potentially affect the structural conformation and stability of proteins significantly, occurred outside the mapped antigenic regions at position 266 (alanine[A] to S) and was also present among strains circulating pre-2009, those of L2. However, it is possible that substitutions in the non-AR could affect the stability of the viral particle or protein assortment specificities, since VP6 serves as an anchor for the outer capsid VP4 and VP7 proteins, where the 260 trimers of VP7 lie directly on top of the VP6 trimers (54). Contact with VP6 is facilitated by the arm-like extensions formed by the VP7 N termini that also form a lattice with other VP7 trimers. This interaction allows the gripping of VP7 to the intermediate VP6 layer and reinforces the integrity of the outer shell (55). Such interactions may drive the selection of particular VP7 and/or VP4 lineages in reassortant strains and explain lineage replacements that may not necessarily be explained exclusively in terms of immune pressure. In a recent analysis, vaccine effectiveness against all G1P[8] strains 3 years after Rotarix introduction in Malawi was 82% (30), suggesting a high degree of protection against atypical DS-1-like G1P[8] strains, given that these G1P[8] strains were detected in randomly selected stool samples collected between 2013 and 2014. Further analysis is under way in order to assess the extent of the spread of the DS-1-like G1P[8] strains and to calculate vaccine effectiveness against various G and P types possessing Wa- and DS-1-like genetic backbones.

In conclusion, genome reassortment and mutation are the major evolutionary mechanisms that influenced the genetic diversity of G1P[8] strains that circulated in Malawi from 1998 to 2014. Atypical DS-1-like G1P[8] strains emerged in 2013 through genome reassortment events between Wa- and DS-1-like human strains that can be traced back to the 1990s in Malawi. Mutations within outer capsid VP7 of Malawian G1P[8] strains compared to RV1 had no impact on the structural conformation of antigenic regions, suggesting little or no effect on the recognition of vaccine-induced antibodies. Thus, the remaining genome segments (non-G or -P) might also play an important role in immune evasion. It is likely that the atypical DS-1-like G1P[8] strains emerged through natural strain evolutionary pressure, which is unrelated to vaccine use. However, the predominance of atypical reassortant DS-1-like G1P[8] strains, which coincided with vaccine introduction, could suggest the positive selection of atypical G1P[8] strains that were undergoing purifying selection. Vaccine effectiveness against such atypical strains needs careful investigation.

## MATERIALS AND METHODS

### Rotavirus strains.

Stool samples collected from children aged <5 years presenting with acute gastroenteritis at QECH in Blantyre, Malawi, from 1998 to 2014 were utilized (27–29). Diarrhea case definition, rotavirus screening, VP4 and VP7 genotyping, and descriptions of strains that circulated in Malawi were reported previously (27–29). In total, 4,888 stool specimens were collected before vaccine introduction (1997 to 2012), whereas 2,109 were collected after vaccine introduction (2013 to 2014). Rotaviruses with G1P[8] outer capsid proteins were the only strains that were detected every year from 1998 to 2015 (comprising 554 strains isolated before and 95 strains isolated after vaccine introduction). Therefore, only G1P[8] strains from each surveillance year, and from samples that had sufficient fecal material for dsRNA reextraction, were selected for further examination. Where available, a single fecal sample from each month of each year was randomly selected for whole-genome sequencing; only samples from which whole-genome data were obtained were included in the analysis. Ethical approval was obtained from the National Health Sciences Research Committee, Lilongwe, Malawi (approval number 867), and the Research Ethics Committee of the University of Liverpool, Liverpool, United Kingdom (approval number 000490).

### Preparation of purified dsRNA and cDNA for rotavirus whole-genome sequencing.

Rotavirus dsRNA was extracted and purified as previously described (17, 56). An additional DNase I treatment step following a lithium chloride precipitation step was included to remove contaminating DNA (Sigma-Aldrich, Dorset, UK). Purified dsRNA was quantified by using a Qubit 3.0 fluorometer (Life Technologies, CA, USA). Sequence-independent cDNA synthesis and PCR amplification procedures described previously (17, 56) were used to amplify cDNA for rotavirus whole genomes from samples with ≥2 ng/μl dsRNA.

### RNA and cDNA library construction and Illumina HiSeq sequencing.

After denaturation of dsRNA at 95°C for 5 min, ScriptSeq RNA-Seq Library Preparation kit V2 was used to generate Illumina sequencing libraries for samples that had <2 ng/μl dsRNA (Illumina Inc., CA, USA). Purified cDNA generated from samples with >2 ng/μl dsRNA was subjected to standard bar-coding and library construction for Illumina sequencing using a Nextera XT DNA library preparation kit (Illumina Inc., CA, USA). Rotavirus VP6-specific quantitative PCR (qPCR) (57) and a 2100 bioanalyzer (Agilent Technologies Inc., CA, USA) were used to perform quality control on the DNA libraries, followed by sequencing using the HiSeq 2000 platform (Illumina Inc., CA, USA) at the Centre for Genomic Research (CGR), University of Liverpool, United Kingdom.

### Sequence assembly and determination of rotavirus genotypes.

Illumina adapter sequences were trimmed from the raw Fastq sequence data by using Cutadapt v1.2.1 (58) and Sickle v1.2 software (59). Complete consensus nucleotide sequences were generated by mapping trimmed Illumina sequence reads to various complete nucleotide sequences of prototype rotavirus genogroup strains by using both *de novo* and reference DNA sequence assembler algorithms implemented in Geneious software v8 (60). Rotavirus genotypes were assigned to each of the 11 genome segments by using the RotaC v2.0 Web-based automated rotavirus genotyping tool (http://rotac.regatools.be/) (7).

### Sequence alignments and maximum likelihood phylogeny construction.

Reference nucleotide sequences for each rotavirus genome segment were retrieved from the rotavirus resource in the GenBank database (61). This was followed by multiple-sequence alignment of the assembled sequences for the study strains by using Muscle v3.8.31 (62), included in MEGA v6.0 (63). Initial phylogenetic trees for each segment were inferred by using the maximum likelihood approach implemented in MEGA by selecting the DNA model that best fit the data according to the corrected Akaike information criterion (AICc), as described previously (12). We used a generalized time-reversible (GTR) model with Gamma (Υ) heterogeneity across nucleotide sites, while the reliability of the branching order and partitioning were assessed by performing 1,000 bootstrap replicates (64).

### Bayesian inference of phylogenies and population dynamics.

Coalescence analyses were performed by using BEAUTi v1.7.5 and BEAST v1.8 (65, 66) with the following parameter specifications: lognormal relaxed (uncorrelated) clock model (67), a constant-size coalescent tree prior, a Hasegawa-Kishino-Yano (HKY85) nucleotide substitution model with estimated base frequencies (68), and a Υ site heterogeneity model with 4 rate categories (69) and a prior mutation rate (μ) of ∼1.0 × 10^−3^ nucleotide substitutions/site/year, as previously reported by Zeller et al. (35). The maximum likelihood trees generated as described above were used as starting trees for the Bayesian analysis in BEAST. We used a GMRF tree prior, which also allows the investigation of the population dynamics, i.e., effective population size (*N*_e_τ) or relative genetic diversity, over time. A total of 200 million Markov chain Monte Carlo (MCMC) iterations were performed and sampled every 40,000th generation. The first 20 million iterations (10% of the total) from the MCMC analysis, burn-in time, were discarded since these iterations may represent states that the chain explored before reaching the equilibrium state of the target distribution. The mean values and 95% HPDs of the mutation rates and the tMRCAs for each rotavirus segment were calculated from the BEAST output by using Tracer v1.6.0 (https://tree.bio.ed.ac.uk/software/tracer/). The MCC tree for each viral segment was generated by using Tree Annotator v2.1.2 (http://beast.bio.ed.ac.uk/treeannotator) and visualized by using FigTree v1.4.2 (http://tree.bio.ed.ac.uk/software/figtree/) and BioPython scripts (70).

### Detection of natural selection in proteins encoded by rotavirus segments.

ORFs that encode both structural and nonstructural proteins were identified and extracted from the multiple-sequence alignments of each rotavirus genomic segment. We used BioPython (70) scripts to extract and manipulate the sequence alignments. The ORF alignments for each protein were then converted to the corresponding codon alignments by using CodonAlign (http://www.hiv.lanl.gov/cgi-bin/CodonAlign). The alignments were then used to calculate the global ratio of synonymous to nonsynonymous substitutions (*dN*/*dS*) for each ORF by using single-likelihood ancestor counting (SLAC) (71) and a fast, unconstrained Bayesian approximation for inferring selection (FUBAR) (72). To identify specific sites under selection in the ORFs, we also used the mixed-effects model of episodic selection (MEME) (73), random-effects likelihood (REL) (74), and fixed-effects likelihood (FEL) (75) methods implemented in DataMonkey, a Web server for the HyPhy package (76). The occurrence of genetic recombination was checked by using GARD (31) and SBP (32). We used the following default significance levels: *P*, Bayes factor, or posterior probability values of 0.1 for SLAC, MEME, and FEL; 0.9 for FUBAR; and 50 for REL. The HKY85 nucleotide substitution model, beta-gamma site-to-site rate variation, and neighbor-joining trees were used for the selection analysis. All analyses were done by using the DataMonkey Web server (71).

### Structure comparison between the outer capsid glycoprotein of RV1 and G1P[8] strains to predict changes in antibody binding.

To investigate the likely impact of amino acid substitutions on anti-RV1 antibody recognition due to mutations that occurred over time within the antigenic regions of Malawian G1P[8] rotaviruses, the structural conformations of the outer capsid glycoprotein of pre- and postvaccine strains were compared to that of RV1. Representative VP7 sequences for each G1P[8] lineage were utilized for protein structure modeling using Modeler version 9.17 (77). Templates were searched in the Protein Data Bank (78), using an integrated Web-based HHpred program (79). The best model with the highest zdope score was selected for analysis from the 100 models that were generated for each sequence.

### Accession number(s).

All complete nucleotide and deduced amino acid sequences generated in this study were deposited in GenBank (61) under accession numbers MG181227 to MG181941.

## Supplementary Material

Supplemental material
