# Supplementary material for: Emergence of Double- and Triple-Gene Reassortant G1P[8] Rotaviruses Possessing a DS-1-Like Backbone after Rotavirus Vaccine Introduction in Malawi
Source: J Virol. 2018 Jan 17;92(3):e01246-17. doi: 10.1128/JVI.01246-17 (PMC5774894; doi:10.1128/JVI.01246-17)
Supplement: Supplemental material [file supp_92_3_e01246-17__index.html]

Supplemental material 

# Emergence of Double- and Triple-Gene Reassortant G1P[8] Rotaviruses Possessing a DS-1-Like Backbone after Rotavirus Vaccine Introduction in Malawi

## Supplemental material

- Supplemental file 1 -

  Table S1 (Whole-genotype constellations of pre- and postvaccine Malawian G1P[8] strains and reference rotaviruses.)

  XLS, 72K
- Supplemental file 2 -

  Fig. S1 (G1P[8] rotavirus strains characterized from stool samples collected from Malawian infants at QECH from 1997 to 2015.)

  Fig. S2 (Phylogenetic analysis of complete ORFs for individual 11 genome segments of Malawian G1P[8] strains compared to reference strains from elsewhere using the maximum-likelihood method.)

  Fig. S3. Bayesian maximum clade credibility (MCC) time tree based on complete nucleotide sequences illustrating lineage replacement within the genome segments encoding nonstructural proteins of the G1P[8] strains that circulated in Malawi from 1998 to 2014.) Fig. S4 (Bayesian MCC time tree based on complete nucleotide sequences of nonstructural proteins for G1P[8] strains from Malawi.)

  PDF, 3.9M
