# Supplementary material for: Emergence of Double- and Triple-Gene Reassortant G1P[8] Rotaviruses Possessing a DS-1-Like Backbone after Rotavirus Vaccine Introduction in Malawi
Source: J Virol. 2018 Jan 17;92(3):e01246-17. doi: 10.1128/JVI.01246-17 (PMC5774894; doi:10.1128/JVI.01246-17)
Supplement: Supplemental material [file JVI.01246-17_zjv003183263s2.pdf]

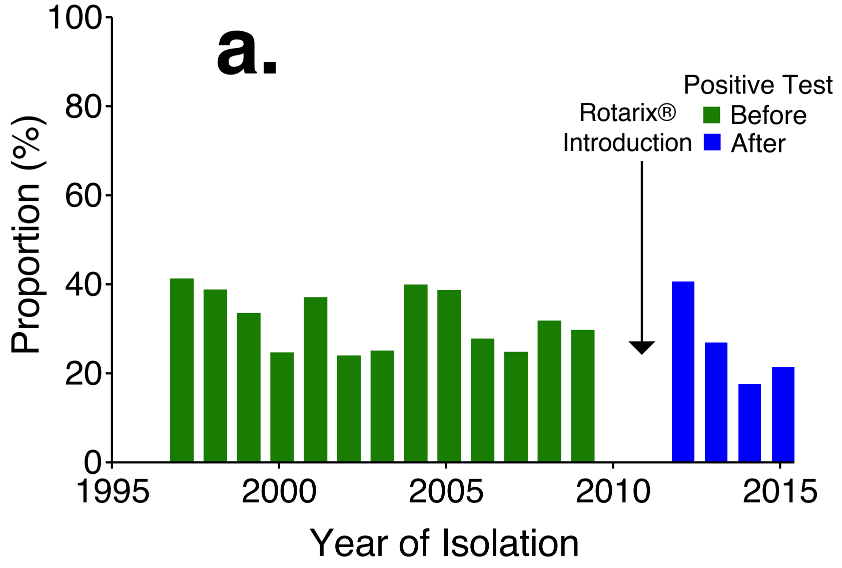
a.
Positive Test
Before
After
Rotarix®
Introduction
Proportion (%)
Year of Isolation
0
20
40
60
80
100
1995
2000
2005
2010
2015

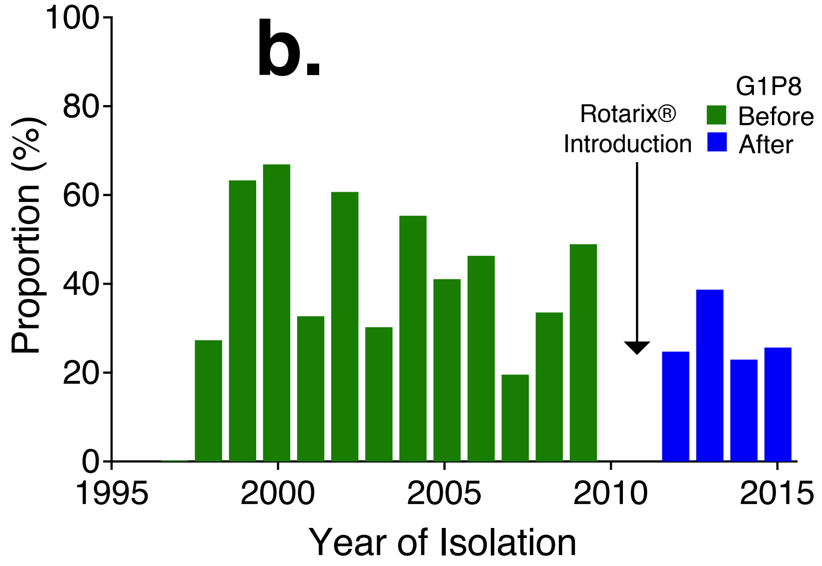
b.
G1P8
Before
After
Rotarix®
Introduction
Proportion (%)
Year of Isolation
0
20
40
60
80
100
1995
2000
2005
2010
2015

C.

YEAR	G12P4	G12P6	G12P8	G1P4	G1P6	G1P8	G2P4	G2P6	G2P8	G3P4	G3P6	G3P8	G4P6	G4P8	G8P4	G8P6	G8P8	G9P4	G9P6	G9P8	TOTAL	TOTAL
**1997**	0	0	0	0	0	0	0	0	0	1	10	28	1	4	8	43	0	0	3	0	98	**98**
**1998**	0	0	0	0	0	55	0	0	0	0	2	61	2	14	22	47	0	0	0	0	203	**203**
**1999**	0	0	0	0	7	94	0	0	0	0	0	4	1	3	1	39	0	0	0	0	149	**149**
**2000**	0	0	0	0	2	78	0	0	0	0	0	0	0	0	1	36	0	0	0	0	117	**117**
**2001**	0	0	0	1	1	25	0	0	0	0	0	0	0	0	29	3	1	0	17	0	77	**77**
**2002**	0	0	0	0	3	26	0	0	0	0	0	0	0	0	3	0	0	0	11	0	43	**43**
**2003**	0	0	0	0	1	9	0	0	0	0	0	0	0	0	0	1	1	0	18	0	30	**30**
**2004**	0	0	0	0	6	54	0	0	0	0	0	0	0	0	0	1	4	0	19	3	87	**87**
**2005**	0	2	0	0	48	40	0	0	0	0	0	0	0	0	13	3	6	0	1	2	115	**115**
**2006**	0	3	0	0	26	41	0	0	0	0	0	0	0	0	2	2	13	0	0	2	89	**89**
**2007**	0	1	4	0	0	6	0	0	0	0	0	0	0	0	15	0	5	0	0	0	31	**31**
**2008**	0	27	4	0	2	31	16	3	1	0	0	0	0	0	0	1	1	0	1	6	93	**93**
**2009**	0	24	1	1	2	56	5	3	0	0	0	0	0	0	14	0	0	0	0	9	115	**115**
**2010**	0	0	0	0	0	0	0	0	0	0	0	0	0	0	0	0	0	0	0	0	0	**0**
**2012**	1	22	9	1	1	39	56	28	2	0	0	0	0	0	0	0	0	0	0	0	159	**159**
**2013**	0	21	14	1	4	60	35	19	1	0	0	0	0	0	0	0	0	1	0	0	156	**156**
**2014**	1	17	0	2	4	20	39	3	1	0	0	0	0	0	0	0	0	0	1	0	88	**88**
**2015**	**0**	**18**	**0**	**1**	**20**	**15**	**2**	**3**	**0**	**0**	**0**	**0**	**0**	**0**	**0**	**0**	**0**	**0**	**0**	**0**	**59**	**59**

Fig. S1. G1P[8] rotavirus strains characterised from stool samples collected from Malawian infants at QECH from 1997 – 2015. **(A)** Proportion of rotavirus-positive samples detected in each year. **(B)** Proportion of G1P[8] strains detected from rotavirus-positive samples. **(C)** Absolute numbers of rotavirus strains characterised from 1997 – 2015.

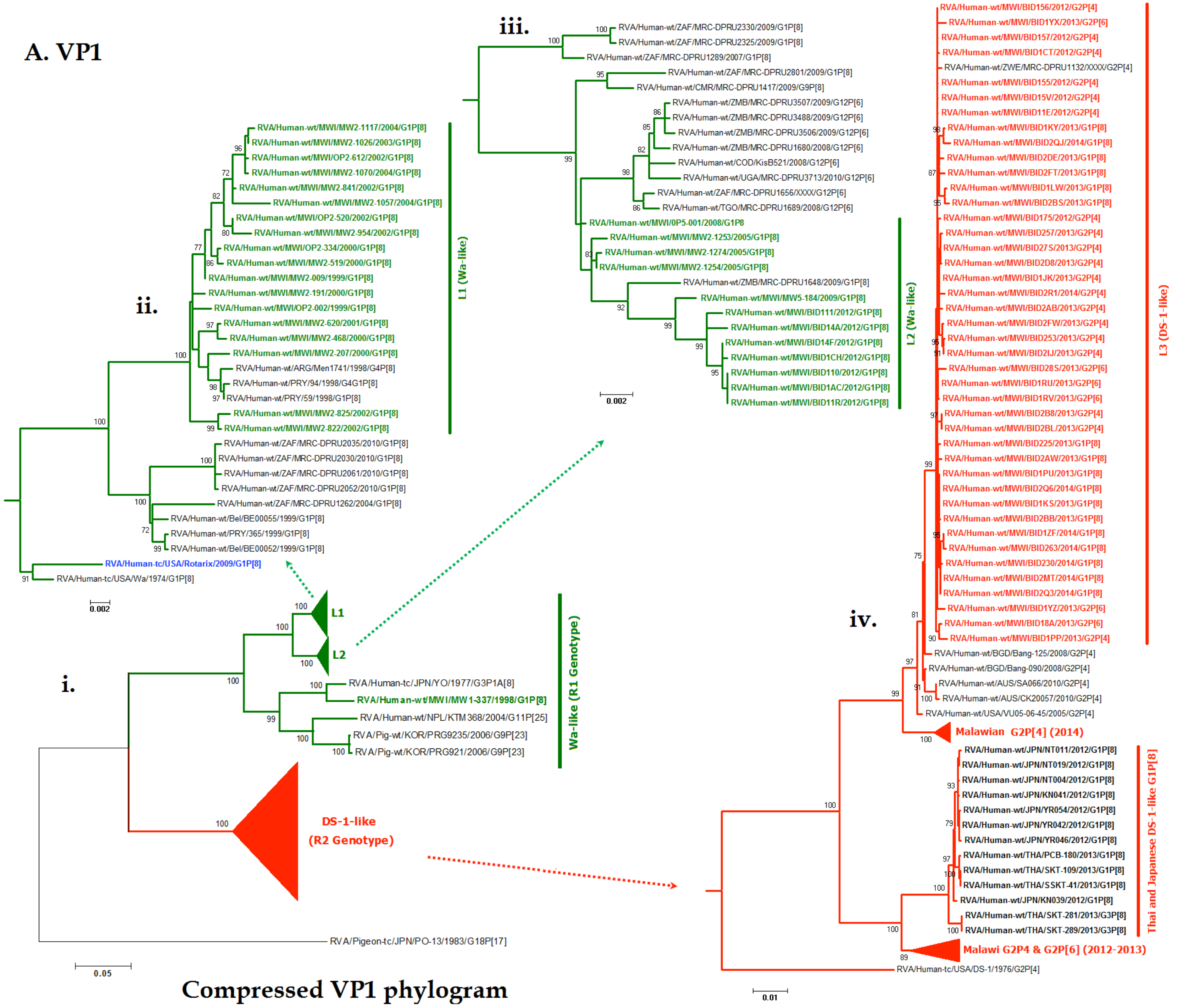
A. VP1
i.
ii.
iii.
iv.
L1
L2
L1 (Wa-like)
L2 (Wa-like)
L3 (DS-1-like)
Wa-like (R1 Genotype)
DS-1-like
(R2 Genotype)
Malawian G2P[4] (2014)
Thai and Japanese DS-1-like G1P[8]
Malawi G2P4 & G2P[6] (2012-2013)
Compressed VP1 phylogram

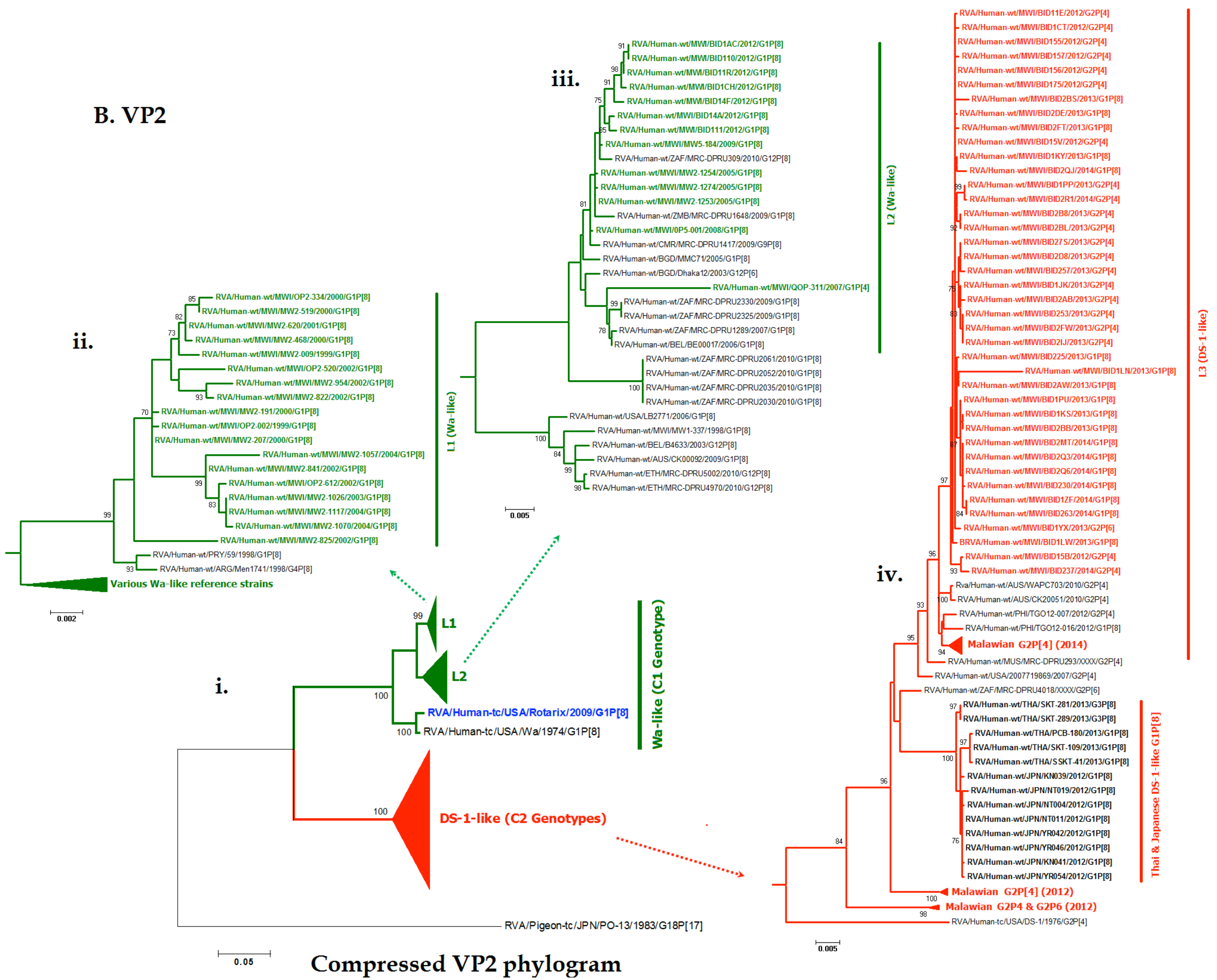
B. VP2
ii.
RVA/Human-wt/MWI/OP2-334/2000/G1P[8]
RVA/Human-wt/MWI/MW2-519/2000/G1P[8]
RVA/Human-wt/MWI/MW2-620/2001/G1P[8]
RVA/Human-wt/MWI/MW2-468/2000/G1P[8]
RVA/Human-wt/MWI/MW2-009/1999/G1P[8]
RVA/Human-wt/MWI/OP2-520/2002/G1P[8]
RVA/Human-wt/MWI/MW2-954/2002/G1P[8]
RVA/Human-wt/MWI/MW2-822/2002/G1P[8]
RVA/Human-wt/MWI/MW2-191/2000/G1P[8]
RVA/Human-wt/MWI/OP2-002/1999/G1P[8]
RVA/Human-wt/MWI/MW2-207/2000/G1P[8]
RVA/Human-wt/MWI/MW2-1057/2004/G1P[8]
RVA/Human-wt/MWI/MW2-841/2002/G1P[8]
RVA/Human-wt/MWI/OP2-612/2002/G1P[8]
RVA/Human-wt/MWI/MW2-1026/2003/G1P[8]
RVA/Human-wt/MWI/MW2-1117/2004/G1P[8]
RVA/Human-wt/MWI/MW2-1070/2004/G1P[8]
RVA/Human-wt/MWI/MW2-825/2002/G1P[8]
RVA/Human-wt/PRY/59/1998/G1P[8]
RVA/Human-wt/ARG/Men1741/1998/G4P[8]
Various Wa-like reference strains
L1 (Wa-like)
0.002
iii.
RVA/Human-wt/MWI/BID1AC/2012/G1P[8]
RVA/Human-wt/MWI/BID110/2012/G1P[8]
RVA/Human-wt/MWI/BID11R/2012/G1P[8]
RVA/Human-wt/MWI/BID1CH/2012/G1P[8]
RVA/Human-wt/MWI/BID14F/2012/G1P[8]
RVA/Human-wt/MWI/BID14A/2012/G1P[8]
RVA/Human-wt/MWI/BID111/2012/G1P[8]
RVA/Human-wt/MWI/MW5-184/2009/G1P[8]
RVA/Human-wt/ZAF/MRC-DPRU309/2010/G12P[8]
RVA/Human-wt/MWI/MW2-1254/2005/G1P[8]
RVA/Human-wt/MWI/MW2-1274/2005/G1P[8]
RVA/Human-wt/MWI/MW2-1253/2005/G1P[8]
RVA/Human-wt/ZMB/MRC-DPRU1648/2009/G1P[8]
RVA/Human-wt/MWI/0P5-001/2008/G1P[8]
RVA/Human-wt/CMR/MRC-DPRU1417/2009/G9P[8]
RVA/Human-wt/BGD/MMC71/2005/G1P[8]
RVA/Human-wt/BGD/Dhaka12/2003/G12P[6]
RVA/Human-wt/MWI/QOP-311/2007/G1P[4]
RVA/Human-wt/ZAF/MRC-DPRU2330/2009/G1P[8]
RVA/Human-wt/ZAF/MRC-DPRU2325/2009/G1P[8]
RVA/Human-wt/ZAF/MRC-DPRU1289/2007/G1P[8]
RVA/Human-wt/BEL/BE00017/2006/G1P[8]
RVA/Human-wt/ZAF/MRC-DPRU2061/2010/G1P[8]
RVA/Human-wt/ZAF/MRC-DPRU2052/2010/G1P[8]
RVA/Human-wt/ZAF/MRC-DPRU2035/2010/G1P[8]
RVA/Human-wt/ZAF/MRC-DPRU2030/2010/G1P[8]
RVA/Human-wt/USA/LB2771/2006/G1P[8]
RVA/Human-wt/MWI/MW1-337/1998/G1P[8]
RVA/Human-wt/BEL/B4633/2003/G12P[8]
RVA/Human-wt/AUS/CK00092/2009/G1P[8]
RVA/Human-wt/ETH/MRC-DPRU5002/2010/G12P[8]
RVA/Human-wt/ETH/MRC-DPRU4970/2010/G12P[8]
L2 (Wa-like)
0.005
i.
L1
L2
RVA/Human-tc/USA/Rotarix/2009/G1P[8]
RVA/Human-tc/USA/Wa/1974/G1P[8]
Wa-like (C1 Genotype)
DS-1-like (C2 Genotypes)
RVA/Pigeon-tc/JPN/PO-13/1983/G18P[17]
0.05
Compressed VP2 phylogram
iv.
RVA/Human-wt/MWI/BID11E/2012/G2P[4]
RVA/Human-wt/MWI/BID1CT/2012/G2P[4]
RVA/Human-wt/MWI/BID155/2012/G2P[4]
RVA/Human-wt/MWI/BID157/2012/G2P[4]
RVA/Human-wt/MWI/BID156/2012/G2P[4]
RVA/Human-wt/MWI/BID175/2012/G2P[4]
RVA/Human-wt/MWI/BID2BS/2013/G1P[8]
RVA/Human-wt/MWI/BID2DE/2013/G1P[8]
RVA/Human-wt/MWI/BID2FT/2013/G1P[8]
RVA/Human-wt/MWI/BID15V/2012/G2P[4]
RVA/Human-wt/MWI/BID1KY/2013/G1P[8]
RVA/Human-wt/MWI/BID2QJ/2014/G1P[8]
RVA/Human-wt/MWI/BID1PP/2013/G2P[4]
RVA/Human-wt/MWI/BID2R1/2014/G2P[4]
RVA/Human-wt/MWI/BID2B8/2013/G2P[4]
RVA/Human-wt/MWI/BID2BL/2013/G2P[4]
RVA/Human-wt/MWI/BID27S/2013/G2P[4]
RVA/Human-wt/MWI/BID2D8/2013/G2P[4]
RVA/Human-wt/MWI/BID257/2013/G2P[4]
RVA/Human-wt/MWI/BID1JK/2013/G2P[4]
RVA/Human-wt/MWI/BID2AB/2013/G2P[4]
RVA/Human-wt/MWI/BID253/2013/G2P[4]
RVA/Human-wt/MWI/BID2FW/2013/G2P[4]
RVA/Human-wt/MWI/BID2IJ/2013/G2P[4]
RVA/Human-wt/MWI/BID225/2013/G1P[8]
RVA/Human-wt/MWI/BID1LN/2013/G1P[8]
RVA/Human-wt/MWI/BID2AW/2013/G1P[8]
RVA/Human-wt/MWI/BID1PU/2013/G1P[8]
RVA/Human-wt/MWI/BID1KS/2013/G1P[8]
RVA/Human-wt/MWI/BID2BB/2013/G1P[8]
RVA/Human-wt/MWI/BID2MT/2014/G1P[8]
RVA/Human-wt/MWI/BID2Q3/2014/G1P[8]
RVA/Human-wt/MWI/BID2Q6/2014/G1P[8]
RVA/Human-wt/MWI/BID230/2014/G1P[8]
RVA/Human-wt/MWI/BID1ZF/2014/G1P[8]
RVA/Human-wt/MWI/BID263/2014/G1P[8]
RVA/Human-wt/MWI/BID1YX/2013/G2P[6]
BRVA/Human-wt/MWI/BID1LW/2013/G1P[8]
RVA/Human-wt/MWI/BID15B/2012/G2P[4]
RVA/Human-wt/MWI/BID237/2014/G2P[4]
Rva/Human-wt/AUS/WAPC703/2010/G2P[4]
RVA/Human-wt/AUS/CK20051/2010/G2P[4]
RVA/Human-wt/PHI/TGO12-007/2012/G2P[4]
RVA/Human-wt/PHI/TGO12-016/2012/G1P[8]
Malawian G2P[4] (2014)
RVA/Human-wt/MUS/MRC-DPRU293/XXXX/G2P[4]
RVA/Human-wt/USA/2007719869/2007/G2P[4]
RVA/Human-wt/ZAF/MRC-DPRU4018/XXXX/G2P[6]
RVA/Human-wt/THA/SKT-281/2013/G3P[8]
RVA/Human-wt/THA/SKT-289/2013/G3P[8]
RVA/Human-wt/THA/PCB-180/2013/G1P[8]
RVA/Human-wt/THA/SKT-109/2013/G1P[8]
RVA/Human-wt/THA/SSKT-41/2013/G1P[8]
RVA/Human-wt/JPN/KN039/2012/G1P[8]
RVA/Human-wt/JPN/NT019/2012/G1P[8]
RVA/Human-wt/JPN/NT004/2012/G1P[8]
RVA/Human-wt/JPN/NT011/2012/G1P[8]
RVA/Human-wt/JPN/YR042/2012/G1P[8]
RVA/Human-wt/JPN/YR046/2012/G1P[8]
RVA/Human-wt/JPN/KN041/2012/G1P[8]
RVA/Human-wt/JPN/YR054/2012/G1P[8]
Malawian G2P[4] (2012)
Malawian G2P4 & G2P6 (2012)
RVA/Human-tc/USA/DS-1/1976/G2P[4]
L3 (DS-1-like)
Thai & Japanese DS-1-like G1P[8]
0.005

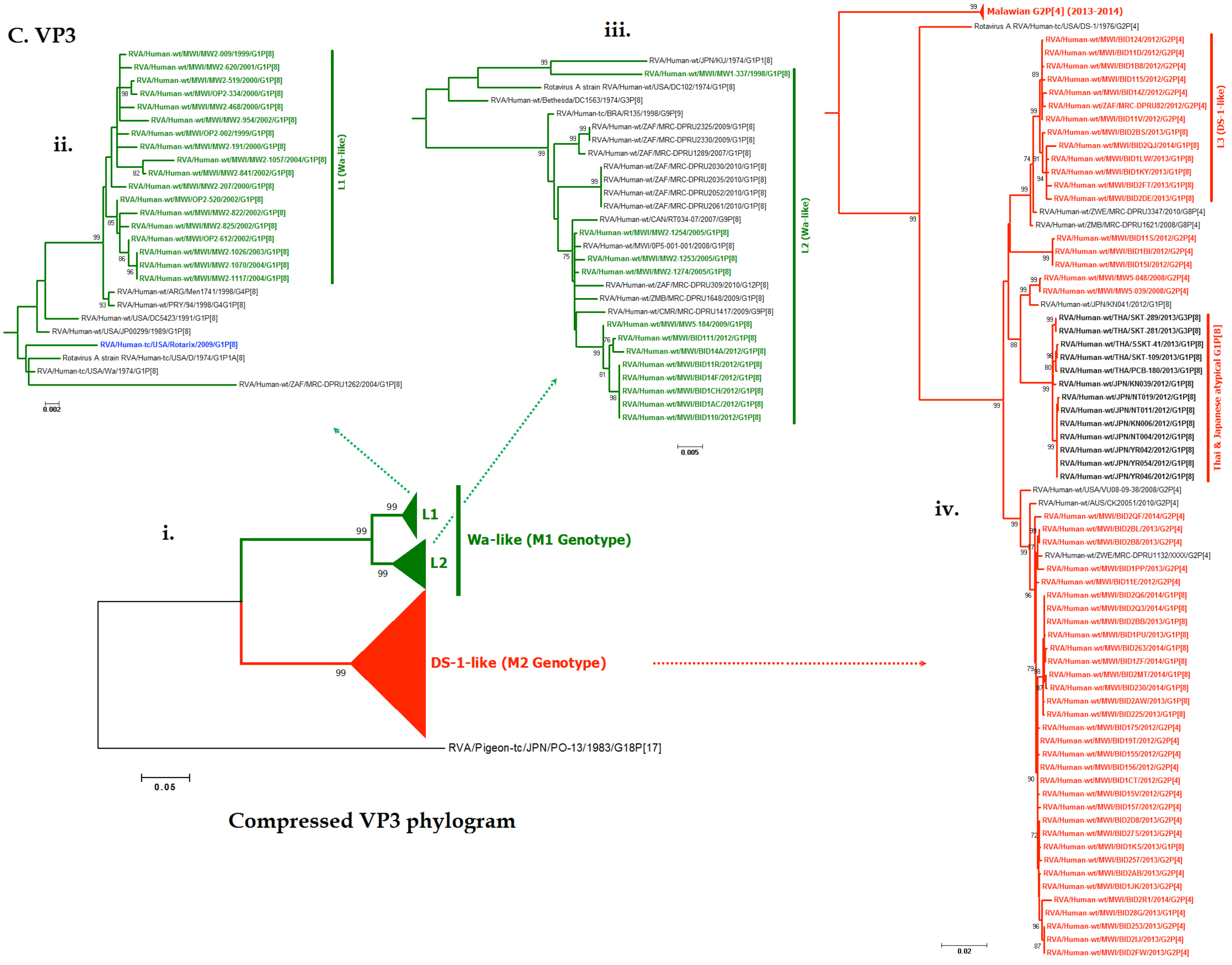
C. VP3
i.
ii.
iii.
iv.
L1
L2
Wa-like (M1 Genotype)
DS-1-like (M2 Genotype)
RVA/Pigeon-tc/JPN/PO-13/1983/G18P[17]
0.05
L1 (Wa-like)
L2 (Wa-like)
L3 (DS-1-like)
Thai & Japanese atypical G1P[8]
Malawian G2P[4] (2013-2014)
Rotavirus A RVA/Human-tc/USA/DS-1/1976/G2P[4]
RVA/Human-tc/USA/Rotarix/2009/G1P[8]
0.002
0.005
0.02
Compressed VP3 phylogram

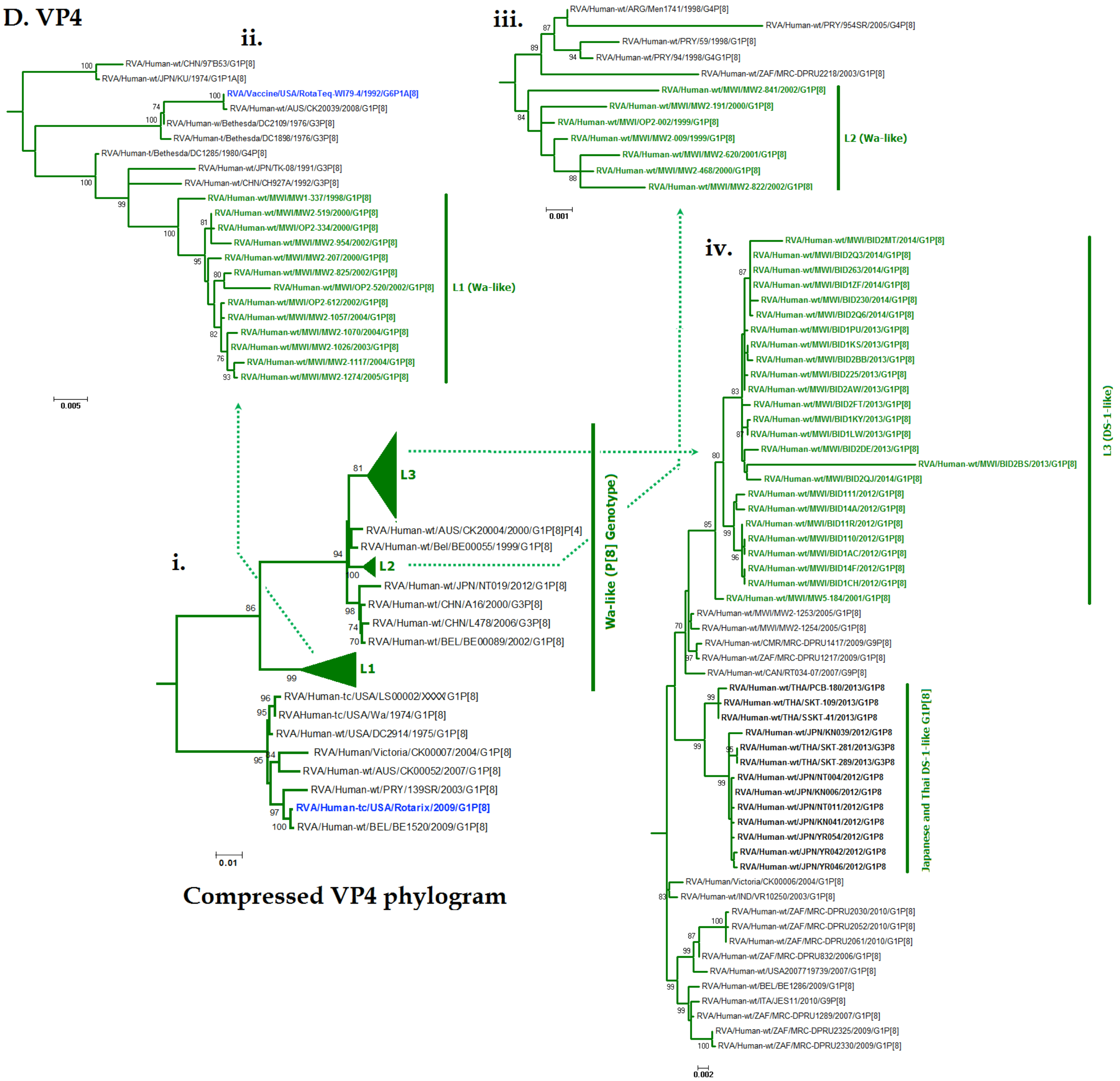

D. VP4
ii.
RVA/Human-wt/CHN/97'B53/G1P[8]
RVA/Human-wt/JPN/KU/1974/G1P1A[8]
RVA/Vaccine/USA/RotaTeq-WI79-4/1992/G6P1A[8]
RVA/Human-wt/AUS/CK20039/2008/G1P[8]
RVA/Human-w/Bethesda/DC2109/1976/G3P[8]
RVA/Human-t/Bethesda/DC1898/1976/G3P[8]
RVA/Human-t/Bethesda/DC1285/1980/G4P[8]
RVA/Human-wt/JPN/TK-08/1991/G3P[8]
RVA/Human-wt/CHN/CH927A/1992/G3P[8]
RVA/Human-wt/MWI/MW1-337/1998/G1P[8]
RVA/Human-wt/MWI/MW2-519/2000/G1P[8]
RVA/Human-wt/MWI/OP2-334/2000/G1P[8]
RVA/Human-wt/MWI/MW2-954/2002/G1P[8]
RVA/Human-wt/MWI/MW2-207/2000/G1P[8]
RVA/Human-wt/MWI/MW2-825/2002/G1P[8]
RVA/Human-wt/MWI/OP2-520/2002/G1P[8]
RVA/Human-wt/MWI/OP2-612/2002/G1P[8]
RVA/Human-wt/MWI/MW2-1057/2004/G1P[8]
RVA/Human-wt/MWI/MW2-1070/2004/G1P[8]
RVA/Human-wt/MWI/MW2-1026/2003/G1P[8]
RVA/Human-wt/MWI/MW2-1117/2004/G1P[8]
RVA/Human-wt/MWI/MW2-1274/2005/G1P[8]
L1 (Wa-like)
0.005
iii.
RVA/Human-wt/ARG/Men1741/1998/G4P[8]
RVA/Human-wt/PRY/954SR/2005/G4P[8]
RVA/Human-wt/PRY/59/1998/G1P[8]
RVA/Human-wt/PRY/94/1998/G4G1P[8]
RVA/Human-wt/ZAF/MRC-DPRU2218/2003/G1P[8]
RVA/Human-wt/MWI/MW2-841/2002/G1P[8]
RVA/Human-wt/MWI/MW2-191/2000/G1P[8]
RVA/Human-wt/MWI/OP2-002/1999/G1P[8]
RVA/Human-wt/MWI/MW2-009/1999/G1P[8]
RVA/Human-wt/MWI/MW2-620/2001/G1P[8]
RVA/Human-wt/MWI/MW2-468/2000/G1P[8]
RVA/Human-wt/MWI/MW2-822/2002/G1P[8]
L2 (Wa-like)
0.001
i.
L3
RVA/Human-wt/AUS/CK20004/2000/G1P[8]P[4]
RVA/Human-wt/Bel/BE00055/1999/G1P[8]
L2
RVA/Human-wt/JPN/NT019/2012/G1P[8]
RVA/Human-wt/CHN/A16/2000/G3P[8]
RVA/Human-wt/CHN/L478/2006/G3P[8]
RVA/Human-wt/BEL/BE00089/2002/G1P[8]
L1
RVA/Human-tc/USA/LS00002/XXXX/G1P[8]
RVAHuman-tc/USA/Wa/1974/G1P[8]
RVA/Human-wt/USA/DC2914/1975/G1P[8]
RVA/Human/Victoria/CK00007/2004/G1P[8]
RVA/Human-wt/AUS/CK00052/2007/G1P[8]
RVA/Human-wt/PRY/139SR/2003/G1P[8]
RVA/Human-tc/USA/Rotarix/2009/G1P[8]
RVA/Human-wt/BEL/BE1520/2009/G1P[8]
Wa-like (P[8] Genotype)
0.01
Compressed VP4 phylogram
iv.
RVA/Human-wt/MWI/BID2MT/2014/G1P[8]
RVA/Human-wt/MWI/BID2Q3/2014/G1P[8]
RVA/Human-wt/MWI/BID263/2014/G1P[8]
RVA/Human-wt/MWI/BID1ZF/2014/G1P[8]
RVA/Human-wt/MWI/BID230/2014/G1P[8]
RVA/Human-wt/MWI/BID2Q6/2014/G1P[8]
RVA/Human-wt/MWI/BID1PU/2013/G1P[8]
RVA/Human-wt/MWI/BID1KS/2013/G1P[8]
RVA/Human-wt/MWI/BID2BB/2013/G1P[8]
RVA/Human-wt/MWI/BID225/2013/G1P[8]
RVA/Human-wt/MWI/BID2AW/2013/G1P[8]
RVA/Human-wt/MWI/BID2FT/2013/G1P[8]
RVA/Human-wt/MWI/BID1KY/2013/G1P[8]
RVA/Human-wt/MWI/BID1LW/2013/G1P[8]
RVA/Human-wt/MWI/BID2DE/2013/G1P[8]
RVA/Human-wt/MWI/BID2BS/2013/G1P[8]
RVA/Human-wt/MWI/BID2QJ/2014/G1P[8]
RVA/Human-wt/MWI/BID111/2012/G1P[8]
RVA/Human-wt/MWI/BID14A/2012/G1P[8]
RVA/Human-wt/MWI/BID11R/2012/G1P[8]
RVA/Human-wt/MWI/BID110/2012/G1P[8]
RVA/Human-wt/MWI/BID1AC/2012/G1P[8]
RVA/Human-wt/MWI/BID14F/2012/G1P[8]
RVA/Human-wt/MWI/BID1CH/2012/G1P[8]
RVA/Human-wt/MWI/MW5-184/2001/G1P[8]
L3 (DS-1-like)
RVA/Human-wt/MWI/MW2-1253/2005/G1P[8]
RVA/Human-wt/MWI/MW2-1254/2005/G1P[8]
RVA/Human-wt/CMR/MRC-DPRU1417/2009/G9P[8]
RVA/Human-wt/ZAF/MRC-DPRU1217/2009/G1P[8]
RVA/Human-wt/CAN/RT034-07/2007/G9P[8]
RVA/Human-wt/THA/PCB-180/2013/G1P8
RVA/Human-wt/THA/SKT-109/2013/G1P8
RVA/Human-wt/THA/SSKT-41/2013/G1P8
RVA/Human-wt/JPN/KN039/2012/G1P8
RVA/Human-wt/THA/SKT-281/2013/G3P8
RVA/Human-wt/THA/SKT-289/2013/G3P8
RVA/Human-wt/JPN/NT004/2012/G1P8
RVA/Human-wt/JPN/KN006/2012/G1P8
RVA/Human-wt/JPN/NT011/2012/G1P8
RVA/Human-wt/JPN/KN041/2012/G1P8
RVA/Human-wt/JPN/YR054/2012/G1P8
RVA/Human-wt/JPN/YR042/2012/G1P8
RVA/Human-wt/JPN/YR046/2012/G1P8
Japanese and Thai DS-1-like G1P[8]
RVA/Human/Victoria/CK00006/2004/G1P[8]
RVA/Human-wt/IND/VR10250/2003/G1P[8]
RVA/Human-wt/ZAF/MRC-DPRU2030/2010/G1P[8]
RVA/Human-wt/ZAF/MRC-DPRU2052/2010/G1P[8]
RVA/Human-wt/ZAF/MRC-DPRU2061/2010/G1P[8]
RVA/Human-wt/ZAF/MRC-DPRU832/2006/G1P[8]
RVA/Human-wt/USA2007719739/2007/G1P[8]
RVA/Human-wt/BEL/BE1286/2009/G1P[8]
RVA/Human-wt/ITA/JES11/2010/G9P[8]
RVA/Human-wt/ZAF/MRC-DPRU1289/2007/G1P[8]
RVA/Human-wt/ZAF/MRC-DPRU2325/2009/G1P[8]
RVA/Human-wt/ZAF/MRC-DPRU2330/2009/G1P[8]
0.002

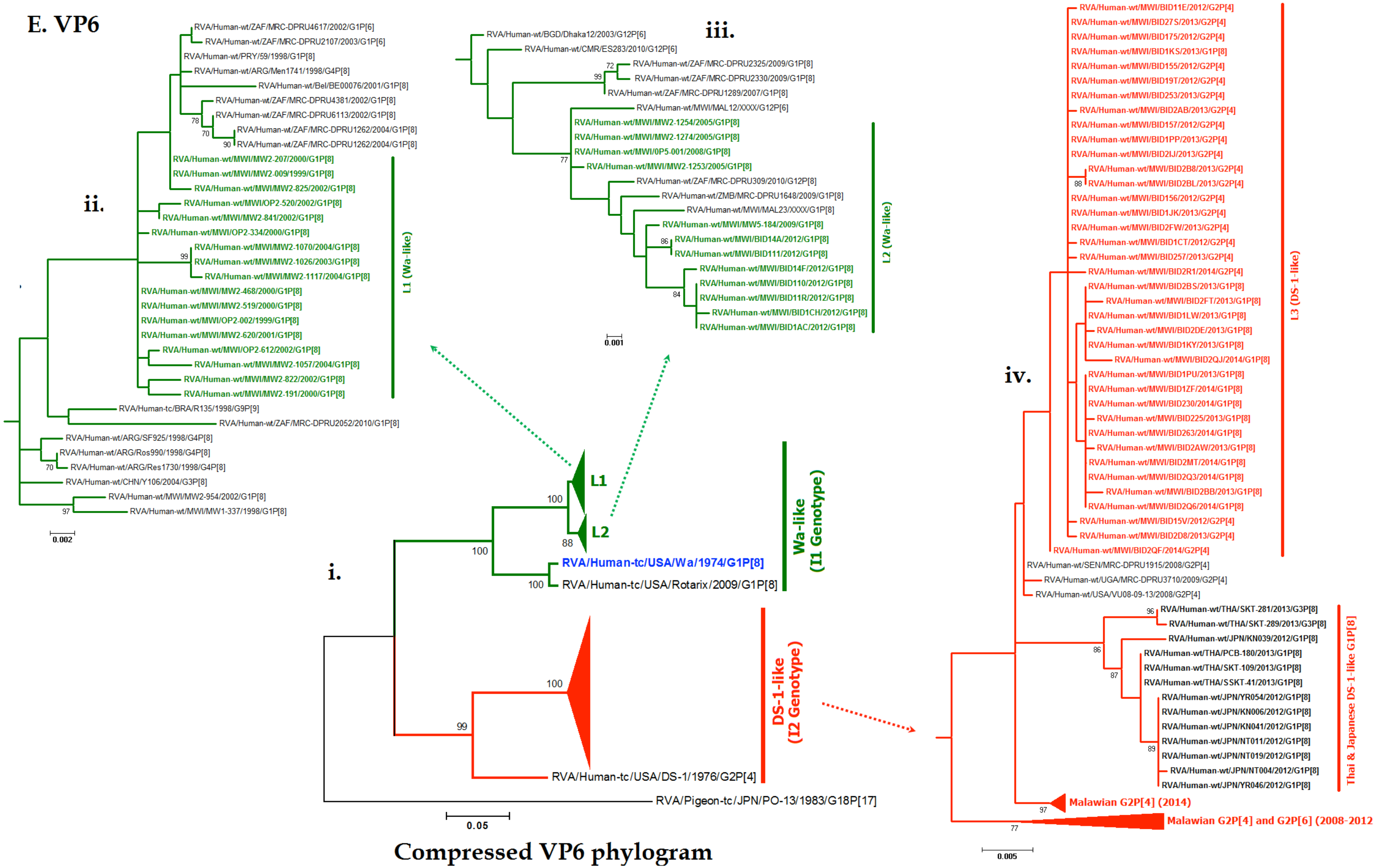
E. VP6
i.
ii.
iii.
iv.
RVA/Human-wt/ZAF/MRC-DPRU4617/2002/G1P[6]
RVA/Human-wt/ZAF/MRC-DPRU2107/2003/G1P[6]
RVA/Human-wt/PRY/59/1998/G1P[8]
RVA/Human-wt/ARG/Men1741/1998/G4P[8]
RVA/Human-wt/Bel/BE00076/2001/G1P[8]
RVA/Human-wt/ZAF/MRC-DPRU4381/2002/G1P[8]
RVA/Human-wt/ZAF/MRC-DPRU6113/2002/G1P[8]
RVA/Human-wt/ZAF/MRC-DPRU1262/2004/G1P[8]
RVA/Human-wt/ZAF/MRC-DPRU1262/2004/G1P[8]
RVA/Human-wt/MWI/MW2-207/2000/G1P[8]
RVA/Human-wt/MWI/MW2-009/1999/G1P[8]
RVA/Human-wt/MWI/MW2-825/2002/G1P[8]
RVA/Human-wt/MWI/OP2-520/2002/G1P[8]
RVA/Human-wt/MWI/MW2-841/2002/G1P[8]
RVA/Human-wt/MWI/OP2-334/2000/G1P[8]
RVA/Human-wt/MWI/MW2-1070/2004/G1P[8]
RVA/Human-wt/MWI/MW2-1026/2003/G1P[8]
RVA/Human-wt/MWI/MW2-1117/2004/G1P[8]
RVA/Human-wt/MWI/MW2-468/2000/G1P[8]
RVA/Human-wt/MWI/MW2-519/2000/G1P[8]
RVA/Human-wt/MWI/OP2-002/1999/G1P[8]
RVA/Human-wt/MWI/MW2-620/2001/G1P[8]
RVA/Human-wt/MWI/OP2-612/2002/G1P[8]
RVA/Human-wt/MWI/MW2-1057/2004/G1P[8]
RVA/Human-wt/MWI/MW2-822/2002/G1P[8]
RVA/Human-wt/MWI/MW2-191/2000/G1P[8]
RVA/Human-tc/BRA/R135/1998/G9P[9]
RVA/Human-wt/ZAF/MRC-DPRU2052/2010/G1P[8]
RVA/Human-wt/ARG/SF925/1998/G4P[8]
RVA/Human-wt/ARG/Ros990/1998/G4P[8]
RVA/Human-wt/ARG/Res1730/1998/G4P[8]
RVA/Human-wt/CHN/Y106/2004/G3P[8]
RVA/Human-wt/MWI/MW2-954/2002/G1P[8]
RVA/Human-wt/MWI/MW1-337/1998/G1P[8]
L1 (Wa-like)
0.002
RVA/Human-wt/BGD/Dhaka12/2003/G12P[6]
RVA/Human-wt/CMR/ES283/2010/G12P[6]
RVA/Human-wt/ZAF/MRC-DPRU2325/2009/G1P[8]
RVA/Human-wt/ZAF/MRC-DPRU2330/2009/G1P[8]
RVA/Human-wt/ZAF/MRC-DPRU1289/2007/G1P[8]
RVA/Human-wt/MWI/MAL12/XXXX/G12P[6]
RVA/Human-wt/MWI/MW2-1254/2005/G1P[8]
RVA/Human-wt/MWI/MW2-1274/2005/G1P[8]
RVA/Human-wt/MWI/OP5-001/2008/G1P[8]
RVA/Human-wt/MWI/MW2-1253/2005/G1P[8]
RVA/Human-wt/ZAF/MRC-DPRU309/2010/G12P[8]
RVA/Human-wt/ZMB/MRC-DPRU1648/2009/G1P[8]
RVA/Human-wt/MWI/MAL23/XXXX/G1P[8]
RVA/Human-wt/MWI/MW5-184/2009/G1P[8]
RVA/Human-wt/MWI/BID14A/2012/G1P[8]
RVA/Human-wt/MWI/BID111/2012/G1P[8]
RVA/Human-wt/MWI/BID14F/2012/G1P[8]
RVA/Human-wt/MWI/BID110/2012/G1P[8]
RVA/Human-wt/MWI/BID11R/2012/G1P[8]
RVA/Human-wt/MWI/BID1CH/2012/G1P[8]
RVA/Human-wt/MWI/BID1AC/2012/G1P[8]
L2 (Wa-like)
0.001
L1
L2
RVA/Human-tc/USA/Wa/1974/G1P[8]
RVA/Human-tc/USA/Rotarix/2009/G1P[8]
Wa-like (I1 Genotype)
RVA/Human-tc/USA/DS-1/1976/G2P[4]
DS-1-like (I2 Genotype)
RVA/Pigeon-tc/JPN/PO-13/1983/G18P[17]
0.05
Compressed VP6 phylogram
RVA/Human-wt/MWI/BID11E/2012/G2P[4]
RVA/Human-wt/MWI/BID27S/2013/G2P[4]
RVA/Human-wt/MWI/BID175/2012/G2P[4]
RVA/Human-wt/MWI/BID1KS/2013/G1P[8]
RVA/Human-wt/MWI/BID155/2012/G2P[4]
RVA/Human-wt/MWI/BID19T/2012/G2P[4]
RVA/Human-wt/MWI/BID253/2013/G2P[4]
RVA/Human-wt/MWI/BID2AB/2013/G2P[4]
RVA/Human-wt/MWI/BID157/2012/G2P[4]
RVA/Human-wt/MWI/BID1PP/2013/G2P[4]
RVA/Human-wt/MWI/BID2IJ/2013/G2P[4]
RVA/Human-wt/MWI/BID2B8/2013/G2P[4]
RVA/Human-wt/MWI/BID2BL/2013/G2P[4]
RVA/Human-wt/MWI/BID156/2012/G2P[4]
RVA/Human-wt/MWI/BID1JK/2013/G2P[4]
RVA/Human-wt/MWI/BID2FW/2013/G2P[4]
RVA/Human-wt/MWI/BID1CT/2012/G2P[4]
RVA/Human-wt/MWI/BID257/2013/G2P[4]
RVA/Human-wt/MWI/BID2R1/2014/G2P[4]
RVA/Human-wt/MWI/BID2BS/2013/G1P[8]
RVA/Human-wt/MWI/BID2FT/2013/G1P[8]
RVA/Human-wt/MWI/BID1LW/2013/G1P[8]
RVA/Human-wt/MWI/BID2DE/2013/G1P[8]
RVA/Human-wt/MWI/BID1KY/2013/G1P[8]
RVA/Human-wt/MWI/BID2QJ/2014/G1P[8]
RVA/Human-wt/MWI/BID1PU/2013/G1P[8]
RVA/Human-wt/MWI/BID1ZF/2014/G1P[8]
RVA/Human-wt/MWI/BID230/2014/G1P[8]
RVA/Human-wt/MWI/BID225/2013/G1P[8]
RVA/Human-wt/MWI/BID263/2014/G1P[8]
RVA/Human-wt/MWI/BID2AW/2013/G1P[8]
RVA/Human-wt/MWI/BID2MT/2014/G1P[8]
RVA/Human-wt/MWI/BID2Q3/2014/G1P[8]
RVA/Human-wt/MWI/BID2BB/2013/G1P[8]
RVA/Human-wt/MWI/BID2Q6/2014/G1P[8]
RVA/Human-wt/MWI/BID15V/2012/G2P[4]
RVA/Human-wt/MWI/BID2D8/2013/G2P[4]
RVA/Human-wt/MWI/BID2QF/2014/G2P[4]
L3 (DS-1-like)
RVA/Human-wt/SEN/MRC-DPRU1915/2008/G2P[4]
RVA/Human-wt/UGA/MRC-DPRU3710/2009/G2P[4]
RVA/Human-wt/USA/VU08-09-13/2008/G2P[4]
RVA/Human-wt/THA/SKT-281/2013/G3P[8]
RVA/Human-wt/THA/SKT-289/2013/G3P[8]
RVA/Human-wt/JPN/KN039/2012/G1P[8]
RVA/Human-wt/THA/PCB-180/2013/G1P[8]
RVA/Human-wt/THA/SKT-109/2013/G1P[8]
RVA/Human-wt/THA/SSKT-41/2013/G1P[8]
RVA/Human-wt/JPN/YR054/2012/G1P[8]
RVA/Human-wt/JPN/KN006/2012/G1P[8]
RVA/Human-wt/JPN/KN041/2012/G1P[8]
RVA/Human-wt/JPN/NT011/2012/G1P[8]
RVA/Human-wt/JPN/NT019/2012/G1P[8]
RVA/Human-wt/JPN/NT004/2012/G1P[8]
RVA/Human-wt/JPN/YR046/2012/G1P[8]
Thai & Japanese DS-1-like G1P[8]
Malawian G2P[4] (2014)
Malawian G2P[4] and G2P[6] (2008-2012
0.005

F. VP7

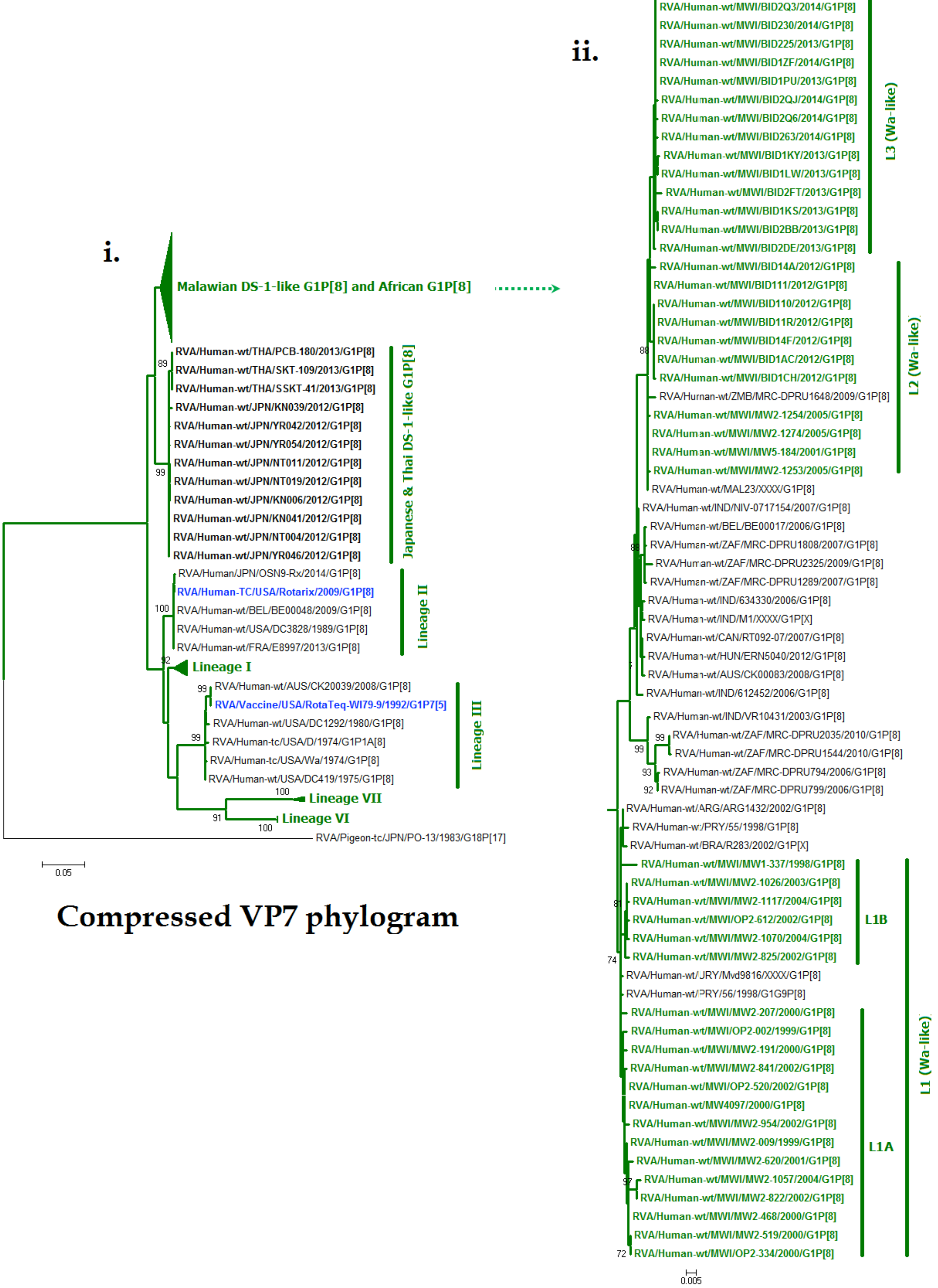

Compressed VP7 phylogram

G. NSP1

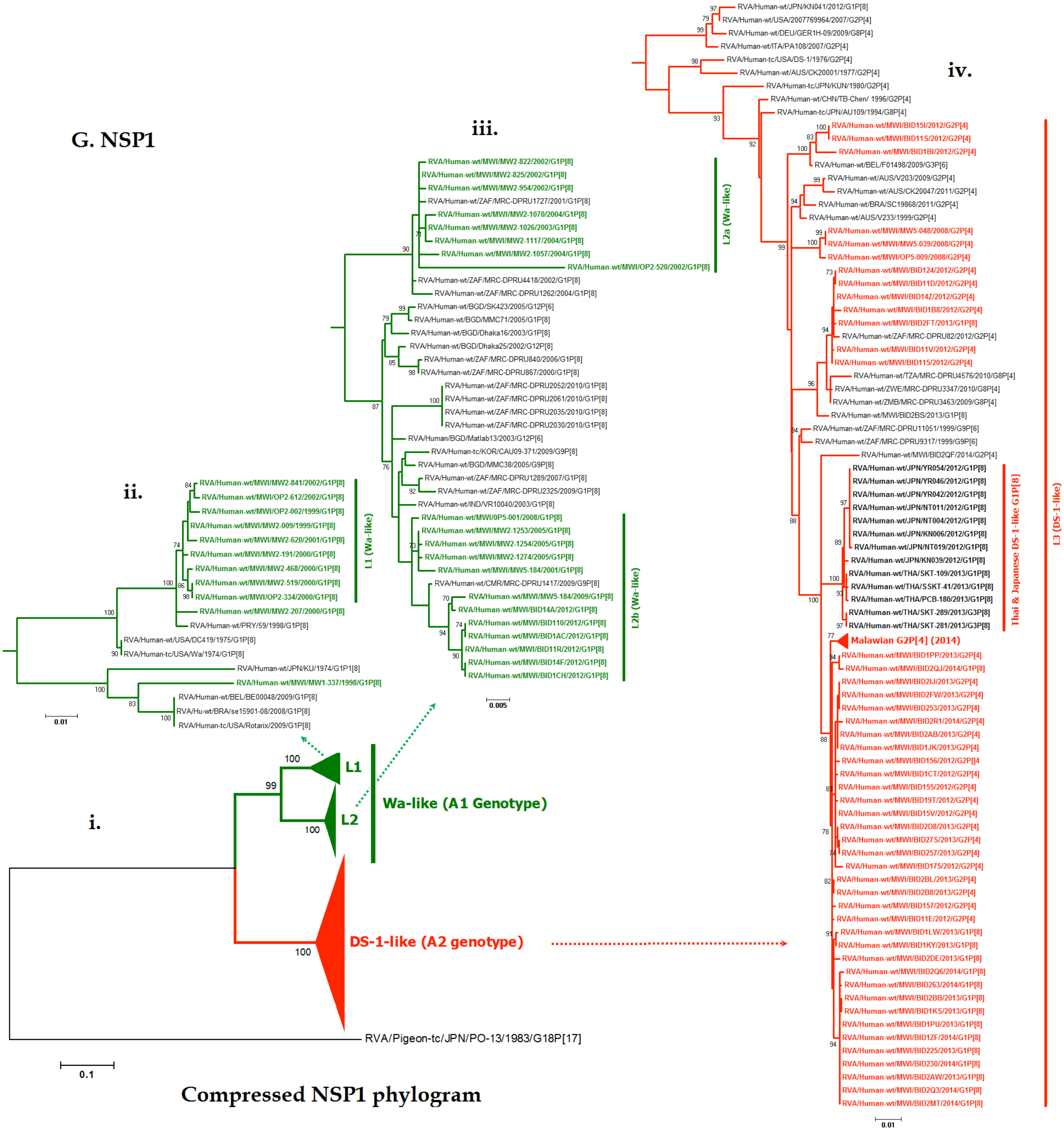

Compressed NSP1 phylogram

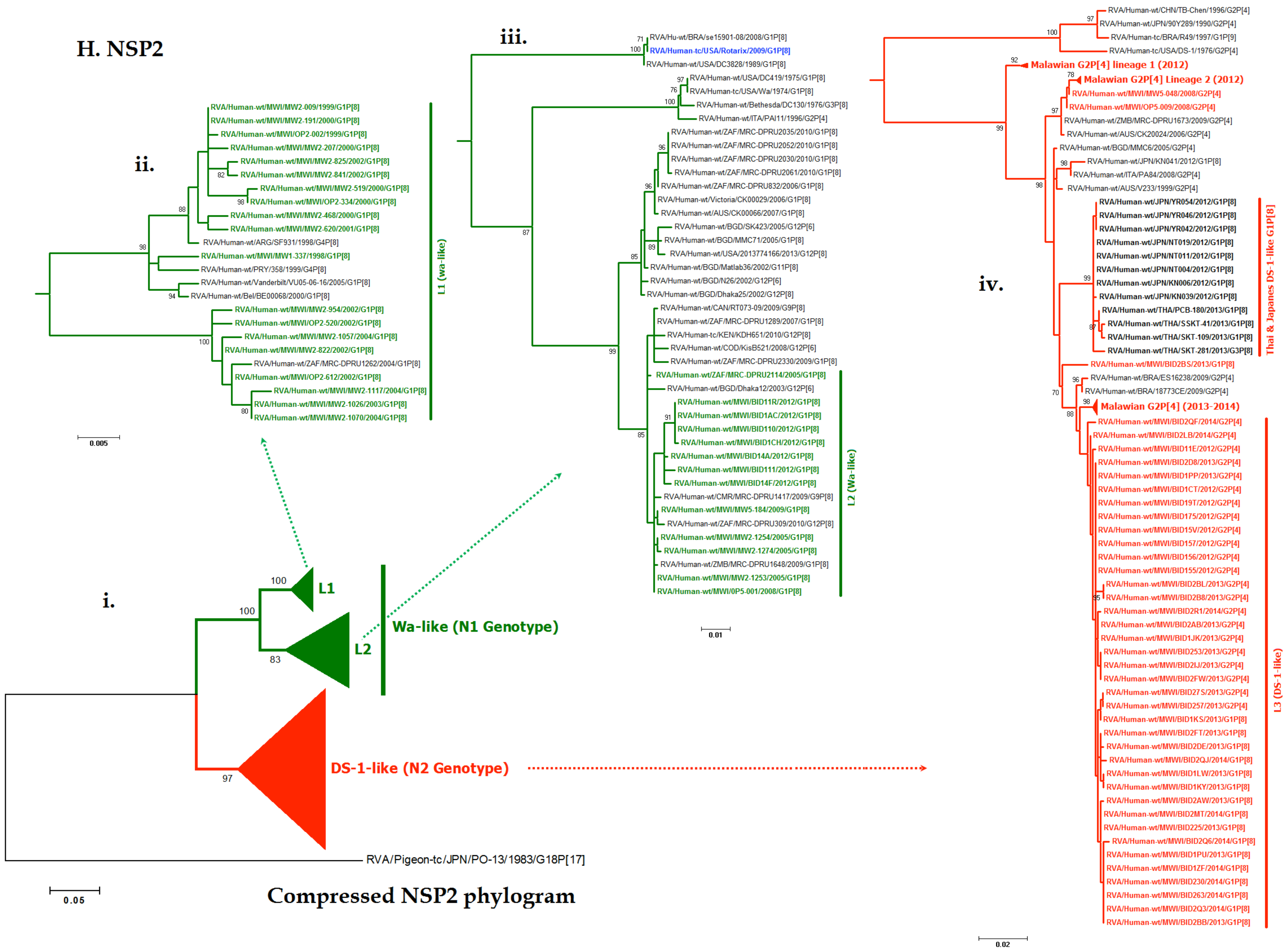
H. NSP2
i.
L1
L2
100
100
83
97
Wa-like (N1 Genotype)
DS-1-like (N2 Genotype)
RVA/Pigeon-tc/JPN/PO-13/1983/G18P[17]
0.05
Compressed NSP2 phylogram
ii.
RVA/Human-wt/MWI/MW2-009/1999/G1P[8]
RVA/Human-wt/MWI/MW2-191/2000/G1P[8]
RVA/Human-wt/MWI/OP2-002/1999/G1P[8]
RVA/Human-wt/MWI/MW2-207/2000/G1P[8]
RVA/Human-wt/MWI/MW2-825/2002/G1P[8]
RVA/Human-wt/MWI/MW2-841/2002/G1P[8]
RVA/Human-wt/MWI/MW2-519/2000/G1P[8]
RVA/Human-wt/MWI/OP2-334/2000/G1P[8]
RVA/Human-wt/MWI/MW2-468/2000/G1P[8]
RVA/Human-wt/MWI/MW2-620/2001/G1P[8]
RVA/Human-wt/ARG/SF931/1998/G4P[8]
RVA/Human-wt/MWI/MW1-337/1998/G1P[8]
RVA/Human-wt/PRY/358/1999/G4P[8]
RVA/Human-wt/Vanderbilt/VU05-06-16/2005/G1P[8]
RVA/Human-wt/Bel/BE00068/2000/G1P[8]
RVA/Human-wt/MWI/MW2-954/2002/G1P[8]
RVA/Human-wt/MWI/OP2-520/2002/G1P[8]
RVA/Human-wt/MWI/MW2-1057/2004/G1P[8]
RVA/Human-wt/MWI/MW2-822/2002/G1P[8]
RVA/Human-wt/ZAF/MRC-DPRU1262/2004/G1P[8]
RVA/Human-wt/MWI/OP2-612/2002/G1P[8]
RVA/Human-wt/MWI/MW2-1117/2004/G1P[8]
RVA/Human-wt/MWI/MW2-1026/2003/G1P[8]
RVA/Human-wt/MWI/MW2-1070/2004/G1P[8]
L1 (wa-like)
0.005
iii.
RVA/Hu-wt/BRA/se15901-08/2008/G1P[8]
RVA/Human-tc/USA/Rotarix/2009/G1P[8]
RVA/Human-wt/USA/DC3828/1989/G1P[8]
RVA/Human-wt/USA/DC419/1975/G1P[8]
RVA/Human-tc/USA/Wa/1974/G1P[8]
RVA/Human-wt/Bethesda/DC130/1976/G3P[8]
RVA/Human-wt/ITA/PAI11/1996/G2P[4]
RVA/Human-wt/ZAF/MRC-DPRU2035/2010/G1P[8]
RVA/Human-wt/ZAF/MRC-DPRU2052/2010/G1P[8]
RVA/Human-wt/ZAF/MRC-DPRU2030/2010/G1P[8]
RVA/Human-wt/ZAF/MRC-DPRU2061/2010/G1P[8]
RVA/Human-wt/ZAF/MRC-DPRU832/2006/G1P[8]
RVA/Human-wt/Victoria/CK00029/2006/G1P[8]
RVA/Human-wt/AUS/CK00066/2007/G1P[8]
RVA/Human-wt/BGD/SK423/2005/G12P[6]
RVA/Human-wt/BGD/MMC71/2005/G1P[8]
RVA/Human-wt/USA/2013774166/2013/G12P[8]
RVA/Human-wt/BGD/Matlab36/2002/G11P[8]
RVA/Human-wt/BGD/N26/2002/G12P[6]
RVA/Human-wt/BGD/Dhaka25/2002/G12P[8]
RVA/Human-wt/CAN/RT073-09/2009/G9P[8]
RVA/Human-wt/ZAF/MRC-DPRU1289/2007/G1P[8]
RVA/Human-tc/KEN/KDH651/2010/G12P[8]
RVA/Human-wt/COD/KisB521/2008/G12P[6]
RVA/Human-wt/ZAF/MRC-DPRU2330/2009/G1P[8]
RVA/Human-wt/ZAF/MRC-DPRU2114/2005/G1P[8]
RVA/Human-wt/BGD/Dhaka12/2003/G12P[6]
RVA/Human-wt/MWI/BID11R/2012/G1P[8]
RVA/Human-wt/MWI/BID1AC/2012/G1P[8]
RVA/Human-wt/MWI/BID110/2012/G1P[8]
RVA/Human-wt/MWI/BID1CH/2012/G1P[8]
RVA/Human-wt/MWI/BID14A/2012/G1P[8]
RVA/Human-wt/MWI/BID111/2012/G1P[8]
RVA/Human-wt/MWI/BID14F/2012/G1P[8]
RVA/Human-wt/CMR/MRC-DPRU1417/2009/G9P[8]
RVA/Human-wt/MWI/MW5-184/2009/G1P[8]
RVA/Human-wt/ZAF/MRC-DPRU309/2010/G12P[8]
RVA/Human-wt/MWI/MW2-1254/2005/G1P[8]
RVA/Human-wt/MWI/MW2-1274/2005/G1P[8]
RVA/Human-wt/ZMB/MRC-DPRU1648/2009/G1P[8]
RVA/Human-wt/MWI/MW2-1253/2005/G1P[8]
RVA/Human-wt/MWI/0P5-001/2008/G1P[8]
L2 (Wa-like)
0.01
iv.
RVA/Human-wt/CHN/TB-Chen/1996/G2P[4]
RVA/Human-wt/JPN/90Y289/1990/G2P[4]
RVA/Human-tc/BRA/R49/1997/G1P[9]
RVA/Human-tc/USA/DS-1/1976/G2P[4]
Malawian G2P[4] lineage 1 (2012)
Malawian G2P[4] Lineage 2 (2012)
RVA/Human-wt/MWI/MW5-048/2008/G2P[4]
RVA/Human-wt/MWI/OP5-009/2008/G2P[4]
RVA/Human-wt/ZMB/MRC-DPRU1673/2009/G2P[4]
RVA/Human-wt/AUS/CK20024/2006/G2P[4]
RVA/Human-wt/BGD/MMC6/2005/G2P[4]
RVA/Human-wt/JPN/KN041/2012/G1P[8]
RVA/Human-wt/ITA/PA84/2008/G2P[4]
RVA/Human-wt/AUS/V233/1999/G2P[4]
RVA/Human-wt/JPN/YR054/2012/G1P[8]
RVA/Human-wt/JPN/YR046/2012/G1P[8]
RVA/Human-wt/JPN/YR042/2012/G1P[8]
RVA/Human-wt/JPN/NT019/2012/G1P[8]
RVA/Human-wt/JPN/NT011/2012/G1P[8]
RVA/Human-wt/JPN/NT004/2012/G1P[8]
RVA/Human-wt/JPN/KN006/2012/G1P[8]
RVA/Human-wt/JPN/KN039/2012/G1P[8]
RVA/Human-wt/THA/PCB-180/2013/G1P[8]
RVA/Human-wt/THA/SSKT-41/2013/G1P[8]
RVA/Human-wt/THA/SKT-109/2013/G1P[8]
RVA/Human-wt/THA/SKT-281/2013/G3P[8]
Thai & Japanes DS-1-like G1P[8]
RVA/Human-wt/MWI/BID2BS/2013/G1P[8]
RVA/Human-wt/BRA/ES16238/2009/G2P[4]
RVA/Human-wt/BRA/18773CE/2009/G2P[4]
Malawian G2P[4] (2013-2014)
RVA/Human-wt/MWI/BID2QF/2014/G2P[4]
RVA/Human-wt/MWI/BID2LB/2014/G2P[4]
RVA/Human-wt/MWI/BID11E/2012/G2P[4]
RVA/Human-wt/MWI/BID2D8/2013/G2P[4]
RVA/Human-wt/MWI/BID1PP/2013/G2P[4]
RVA/Human-wt/MWI/BID1CT/2012/G2P[4]
RVA/Human-wt/MWI/BID19T/2012/G2P[4]
RVA/Human-wt/MWI/BID175/2012/G2P[4]
RVA/Human-wt/MWI/BID15V/2012/G2P[4]
RVA/Human-wt/MWI/BID157/2012/G2P[4]
RVA/Human-wt/MWI/BID156/2012/G2P[4]
RVA/Human-wt/MWI/BID155/2012/G2P[4]
RVA/Human-wt/MWI/BID2BL/2013/G2P[4]
RVA/Human-wt/MWI/BID2B8/2013/G2P[4]
RVA/Human-wt/MWI/BID2R1/2014/G2P[4]
RVA/Human-wt/MWI/BID2AB/2013/G2P[4]
RVA/Human-wt/MWI/BID1JK/2013/G2P[4]
RVA/Human-wt/MWI/BID253/2013/G2P[4]
RVA/Human-wt/MWI/BID2IJ/2013/G2P[4]
RVA/Human-wt/MWI/BID2FW/2013/G2P[4]
RVA/Human-wt/MWI/BID27S/2013/G2P[4]
RVA/Human-wt/MWI/BID257/2013/G2P[4]
RVA/Human-wt/MWI/BID1KS/2013/G1P[8]
RVA/Human-wt/MWI/BID2FT/2013/G1P[8]
RVA/Human-wt/MWI/BID2DE/2013/G1P[8]
RVA/Human-wt/MWI/BID2QJ/2014/G1P[8]
RVA/Human-wt/MWI/BID1LW/2013/G1P[8]
RVA/Human-wt/MWI/BID1KY/2013/G1P[8]
RVA/Human-wt/MWI/BID2AW/2013/G1P[8]
RVA/Human-wt/MWI/BID2MT/2014/G1P[8]
RVA/Human-wt/MWI/BID225/2013/G1P[8]
RVA/Human-wt/MWI/BID2Q6/2014/G1P[8]
RVA/Human-wt/MWI/BID1PU/2013/G1P[8]
RVA/Human-wt/MWI/BID1ZF/2014/G1P[8]
RVA/Human-wt/MWI/BID230/2014/G1P[8]
RVA/Human-wt/MWI/BID263/2014/G1P[8]
RVA/Human-wt/MWI/BID2Q3/2014/G1P[8]
RVA/Human-wt/MWI/BID2BB/2013/G1P[8]
L3 (DS-1-like)
0.02

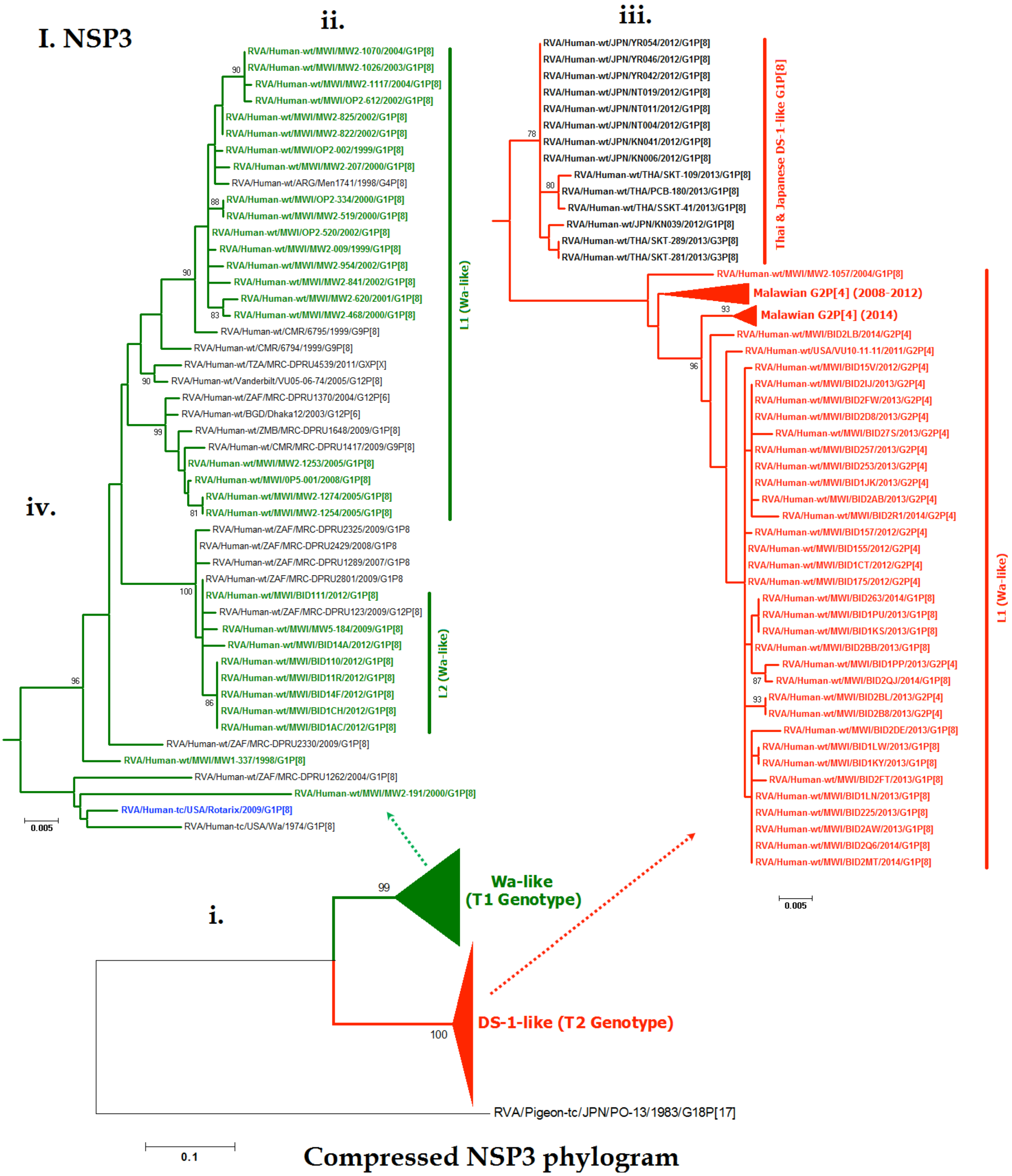

I. NSP3
ii.
RVA/Human-wt/MWI/MW2-1070/2004/G1P[8]
RVA/Human-wt/MWI/MW2-1026/2003/G1P[8]
RVA/Human-wt/MWI/MW2-1117/2004/G1P[8]
RVA/Human-wt/MWI/OP2-612/2002/G1P[8]
RVA/Human-wt/MWI/MW2-825/2002/G1P[8]
RVA/Human-wt/MWI/MW2-822/2002/G1P[8]
RVA/Human-wt/MWI/OP2-002/1999/G1P[8]
RVA/Human-wt/MWI/MW2-207/2000/G1P[8]
RVA/Human-wt/ARG/Men1741/1998/G4P[8]
RVA/Human-wt/MWI/OP2-334/2000/G1P[8]
RVA/Human-wt/MWI/MW2-519/2000/G1P[8]
RVA/Human-wt/MWI/OP2-520/2002/G1P[8]
RVA/Human-wt/MWI/MW2-009/1999/G1P[8]
RVA/Human-wt/MWI/MW2-954/2002/G1P[8]
RVA/Human-wt/MWI/MW2-841/2002/G1P[8]
RVA/Human-wt/MWI/MW2-620/2001/G1P[8]
RVA/Human-wt/MWI/MW2-468/2000/G1P[8]
RVA/Human-wt/CMR/6795/1999/G9P[8]
RVA/Human-wt/CMR/6794/1999/G9P[8]
RVA/Human-wt/TZA/MRC-DPRU4539/2011/GXP[X]
RVA/Human-wt/Vanderbilt/VU05-06-74/2005/G12P[8]
RVA/Human-wt/ZAF/MRC-DPRU1370/2004/G12P[6]
RVA/Human-wt/BGD/Dhaka12/2003/G12P[6]
RVA/Human-wt/ZMB/MRC-DPRU1648/2009/G1P[8]
RVA/Human-wt/CMR/MRC-DPRU1417/2009/G9P[8]
RVA/Human-wt/MWI/MW2-1253/2005/G1P[8]
RVA/Human-wt/MWI/0P5-001/2008/G1P[8]
RVA/Human-wt/MWI/MW2-1274/2005/G1P[8]
RVA/Human-wt/MWI/MW2-1254/2005/G1P[8]
L1 (Wa-like)
RVA/Human-wt/ZAF/MRC-DPRU2325/2009/G1P8
RVA/Human-wt/ZAF/MRC-DPRU2429/2008/G1P8
RVA/Human-wt/ZAF/MRC-DPRU1289/2007/G1P8
RVA/Human-wt/ZAF/MRC-DPRU2801/2009/G1P8
RVA/Human-wt/MWI/BID111/2012/G1P[8]
RVA/Human-wt/ZAF/MRC-DPRU123/2009/G12P[8]
RVA/Human-wt/MWI/MW5-184/2009/G1P[8]
RVA/Human-wt/MWI/BID14A/2012/G1P[8]
RVA/Human-wt/MWI/BID110/2012/G1P[8]
RVA/Human-wt/MWI/BID11R/2012/G1P[8]
RVA/Human-wt/MWI/BID14F/2012/G1P[8]
RVA/Human-wt/MWI/BID1CH/2012/G1P[8]
RVA/Human-wt/MWI/BID1AC/2012/G1P[8]
L2 (Wa-like)
RVA/Human-wt/ZAF/MRC-DPRU2330/2009/G1P[8]
RVA/Human-wt/MWI/MW1-337/1998/G1P[8]
RVA/Human-wt/ZAF/MRC-DPRU1262/2004/G1P[8]
RVA/Human-wt/MWI/MW2-191/2000/G1P[8]
RVA/Human-tc/USA/Rotarix/2009/G1P[8]
RVA/Human-tc/USA/Wa/1974/G1P[8]
iv.
0.005
iii.
RVA/Human-wt/JPN/YR054/2012/G1P[8]
RVA/Human-wt/JPN/YR046/2012/G1P[8]
RVA/Human-wt/JPN/YR042/2012/G1P[8]
RVA/Human-wt/JPN/NT019/2012/G1P[8]
RVA/Human-wt/JPN/NT011/2012/G1P[8]
RVA/Human-wt/JPN/NT004/2012/G1P[8]
RVA/Human-wt/JPN/KN041/2012/G1P[8]
RVA/Human-wt/JPN/KN006/2012/G1P[8]
RVA/Human-wt/THA/SKT-109/2013/G1P[8]
RVA/Human-wt/THA/PCB-180/2013/G1P[8]
RVA/Human-wt/THA/SSKT-41/2013/G1P[8]
RVA/Human-wt/JPN/KN039/2012/G1P[8]
RVA/Human-wt/THA/SKT-289/2013/G3P[8]
RVA/Human-wt/THA/SKT-281/2013/G3P[8]
Thai & Japanese DS-1-like G1P[8]
RVA/Human-wt/MWI/MW2-1057/2004/G1P[8]
Malawian G2P[4] (2008-2012)
Malawian G2P[4] (2014)
RVA/Human-wt/MWI/BID2LB/2014/G2P[4]
RVA/Human-wt/USA/VU10-11-11/2011/G2P[4]
RVA/Human-wt/MWI/BID15V/2012/G2P[4]
RVA/Human-wt/MWI/BID2IJ/2013/G2P[4]
RVA/Human-wt/MWI/BID2FW/2013/G2P[4]
RVA/Human-wt/MWI/BID2D8/2013/G2P[4]
RVA/Human-wt/MWI/BID27S/2013/G2P[4]
RVA/Human-wt/MWI/BID257/2013/G2P[4]
RVA/Human-wt/MWI/BID253/2013/G2P[4]
RVA/Human-wt/MWI/BID1JK/2013/G2P[4]
RVA/Human-wt/MWI/BID2AB/2013/G2P[4]
RVA/Human-wt/MWI/BID2R1/2014/G2P[4]
RVA/Human-wt/MWI/BID157/2012/G2P[4]
RVA/Human-wt/MWI/BID155/2012/G2P[4]
RVA/Human-wt/MWI/BID1CT/2012/G2P[4]
RVA/Human-wt/MWI/BID175/2012/G2P[4]
RVA/Human-wt/MWI/BID263/2014/G1P[8]
RVA/Human-wt/MWI/BID1PU/2013/G1P[8]
RVA/Human-wt/MWI/BID1KS/2013/G1P[8]
RVA/Human-wt/MWI/BID2BB/2013/G1P[8]
RVA/Human-wt/MWI/BID1PP/2013/G2P[4]
RVA/Human-wt/MWI/BID2QJ/2014/G1P[8]
RVA/Human-wt/MWI/BID2BL/2013/G2P[4]
RVA/Human-wt/MWI/BID2B8/2013/G2P[4]
RVA/Human-wt/MWI/BID2DE/2013/G1P[8]
RVA/Human-wt/MWI/BID1LW/2013/G1P[8]
RVA/Human-wt/MWI/BID1KY/2013/G1P[8]
RVA/Human-wt/MWI/BID2FT/2013/G1P[8]
RVA/Human-wt/MWI/BID1LN/2013/G1P[8]
RVA/Human-wt/MWI/BID225/2013/G1P[8]
RVA/Human-wt/MWI/BID2AW/2013/G1P[8]
RVA/Human-wt/MWI/BID2Q6/2014/G1P[8]
RVA/Human-wt/MWI/BID2MT/2014/G1P[8]
L1 (Wa-like)
0.005
i.
Wa-like
(T1 Genotype)
DS-1-like (T2 Genotype)
RVA/Pigeon-tc/JPN/PO-13/1983/G18P[17]
0.1
Compressed NSP3 phylogram

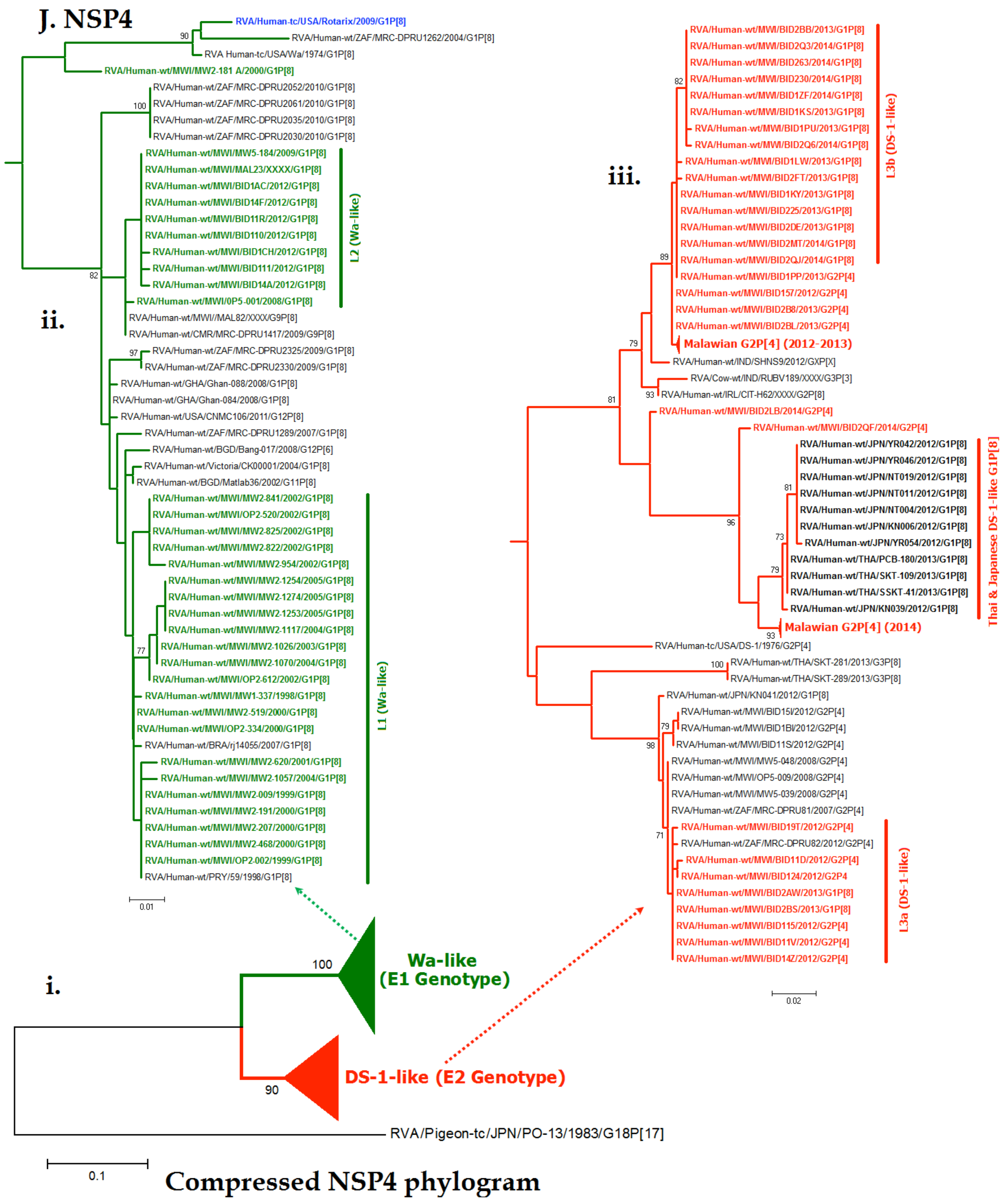
J. NSP4
RVA/Human-tc/USA/Rotarix/2009/G1P[8]
RVA/Human-wt/ZAF/MRC-DPRU1262/2004/G1P[8]
RVA Human-tc/USA/Wa/1974/G1P[8]
RVA/Human-wt/MWI/MW2-181 A/2000/G1P[8]
RVA/Human-wt/ZAF/MRC-DPRU2052/2010/G1P[8]
RVA/Human-wt/ZAF/MRC-DPRU2061/2010/G1P[8]
RVA/Human-wt/ZAF/MRC-DPRU2035/2010/G1P[8]
RVA/Human-wt/ZAF/MRC-DPRU2030/2010/G1P[8]
RVA/Human-wt/MWI/MW5-184/2009/G1P[8]
RVA/Human-wt/MWI/MAL23/XXXX/G1P[8]
RVA/Human-wt/MWI/BID1AC/2012/G1P[8]
RVA/Human-wt/MWI/BID14F/2012/G1P[8]
RVA/Human-wt/MWI/BID11R/2012/G1P[8]
RVA/Human-wt/MWI/BID110/2012/G1P[8]
RVA/Human-wt/MWI/BID1CH/2012/G1P[8]
RVA/Human-wt/MWI/BID111/2012/G1P[8]
RVA/Human-wt/MWI/BID14A/2012/G1P[8]
RVA/Human-wt/MWI/0P5-001/2008/G1P[8]
RVA/Human-wt/MWI//MAL82/XXXX/G9P[8]
RVA/Human-wt/CMR/MRC-DPRU1417/2009/G9P[8]
RVA/Human-wt/ZAF/MRC-DPRU2325/2009/G1P[8]
RVA/Human-wt/ZAF/MRC-DPRU2330/2009/G1P[8]
RVA/Human-wt/GHA/Ghan-088/2008/G1P[8]
RVA/Human-wt/GHA/Ghan-084/2008/G1P[8]
RVA/Human-wt/USA/CNMC106/2011/G12P[8]
RVA/Human-wt/ZAF/MRC-DPRU1289/2007/G1P[8]
RVA/Human-wt/BGD/Bang-017/2008/G12P[6]
RVA/Human-wt/Victoria/CK00001/2004/G1P[8]
RVA/Human-wt/BGD/Matlab36/2002/G11P[8]
RVA/Human-wt/MWI/MW2-841/2002/G1P[8]
RVA/Human-wt/MWI/OP2-520/2002/G1P[8]
RVA/Human-wt/MWI/MW2-825/2002/G1P[8]
RVA/Human-wt/MWI/MW2-822/2002/G1P[8]
RVA/Human-wt/MWI/MW2-954/2002/G1P[8]
RVA/Human-wt/MWI/MW2-1254/2005/G1P[8]
RVA/Human-wt/MWI/MW2-1274/2005/G1P[8]
RVA/Human-wt/MWI/MW2-1253/2005/G1P[8]
RVA/Human-wt/MWI/MW2-1117/2004/G1P[8]
RVA/Human-wt/MWI/MW2-1026/2003/G1P[8]
RVA/Human-wt/MWI/MW2-1070/2004/G1P[8]
RVA/Human-wt/MWI/OP2-612/2002/G1P[8]
RVA/Human-wt/MWI/MW1-337/1998/G1P[8]
RVA/Human-wt/MWI/MW2-519/2000/G1P[8]
RVA/Human-wt/MWI/OP2-334/2000/G1P[8]
RVA/Human-wt/BRA/rj14055/2007/G1P[8]
RVA/Human-wt/MWI/MW2-620/2001/G1P[8]
RVA/Human-wt/MWI/MW2-1057/2004/G1P[8]
RVA/Human-wt/MWI/MW2-009/1999/G1P[8]
RVA/Human-wt/MWI/MW2-191/2000/G1P[8]
RVA/Human-wt/MWI/MW2-207/2000/G1P[8]
RVA/Human-wt/MWI/MW2-468/2000/G1P[8]
RVA/Human-wt/MWI/OP2-002/1999/G1P[8]
RVA/Human-wt/PRY/59/1998/G1P[8]
L2 (Wa-like)
L1 (Wa-like)
ii.
0.01
RVA/Human-wt/MWI/BID2BB/2013/G1P[8]
RVA/Human-wt/MWI/BID2Q3/2014/G1P[8]
RVA/Human-wt/MWI/BID263/2014/G1P[8]
RVA/Human-wt/MWI/BID230/2014/G1P[8]
RVA/Human-wt/MWI/BID1ZF/2014/G1P[8]
RVA/Human-wt/MWI/BID1KS/2013/G1P[8]
RVA/Human-wt/MWI/BID1PU/2013/G1P[8]
RVA/Human-wt/MWI/BID2Q6/2014/G1P[8]
RVA/Human-wt/MWI/BID1LW/2013/G1P[8]
RVA/Human-wt/MWI/BID2FT/2013/G1P[8]
RVA/Human-wt/MWI/BID1KY/2013/G1P[8]
RVA/Human-wt/MWI/BID225/2013/G1P[8]
RVA/Human-wt/MWI/BID2DE/2013/G1P[8]
RVA/Human-wt/MWI/BID2MT/2014/G1P[8]
RVA/Human-wt/MWI/BID2QJ/2014/G1P[8]
RVA/Human-wt/MWI/BID1PP/2013/G2P[4]
RVA/Human-wt/MWI/BID157/2012/G2P[4]
RVA/Human-wt/MWI/BID2B8/2013/G2P[4]
RVA/Human-wt/MWI/BID2BL/2013/G2P[4]
Malawian G2P[4] (2012-2013)
RVA/Human-wt/IND/SHNS9/2012/GXP[X]
RVA/Cow-wt/IND/RUBV189/XXXX/G3P[3]
RVA/Human-wt/IRL/CIT-H62/XXXX/G2P[8]
RVA/Human-wt/MWI/BID2LB/2014/G2P[4]
RVA/Human-wt/MWI/BID2QF/2014/G2P[4]
RVA/Human-wt/JPN/YR042/2012/G1P[8]
RVA/Human-wt/JPN/YR046/2012/G1P[8]
RVA/Human-wt/JPN/NT019/2012/G1P[8]
RVA/Human-wt/JPN/NT011/2012/G1P[8]
RVA/Human-wt/JPN/NT004/2012/G1P[8]
RVA/Human-wt/JPN/KN006/2012/G1P[8]
RVA/Human-wt/JPN/YR054/2012/G1P[8]
RVA/Human-wt/THA/PCB-180/2013/G1P[8]
RVA/Human-wt/THA/SKT-109/2013/G1P[8]
RVA/Human-wt/THA/SSKT-41/2013/G1P[8]
RVA/Human-wt/JPN/KN039/2012/G1P[8]
Malawian G2P[4] (2014)
RVA/Human-tc/USA/DS-1/1976/G2P[4]
RVA/Human-wt/THA/SKT-281/2013/G3P[8]
RVA/Human-wt/THA/SKT-289/2013/G3P[8]
RVA/Human-wt/JPN/KN041/2012/G1P[8]
RVA/Human-wt/MWI/BID15I/2012/G2P[4]
RVA/Human-wt/MWI/BID1BI/2012/G2P[4]
RVA/Human-wt/MWI/BID11S/2012/G2P[4]
RVA/Human-wt/MWI/MW5-048/2008/G2P[4]
RVA/Human-wt/MWI/OP5-009/2008/G2P[4]
RVA/Human-wt/MWI/MW5-039/2008/G2P[4]
RVA/Human-wt/ZAF/MRC-DPRU81/2007/G2P[4]
RVA/Human-wt/MWI/BID19T/2012/G2P[4]
RVA/Human-wt/ZAF/MRC-DPRU82/2012/G2P[4]
RVA/Human-wt/MWI/BID11D/2012/G2P[4]
RVA/Human-wt/MWI/BID124/2012/G2P4
RVA/Human-wt/MWI/BID2AW/2013/G1P[8]
RVA/Human-wt/MWI/BID2BS/2013/G1P[8]
RVA/Human-wt/MWI/BID115/2012/G2P[4]
RVA/Human-wt/MWI/BID11V/2012/G2P[4]
RVA/Human-wt/MWI/BID14Z/2012/G2P[4]
L3b (DS-1-like)
Thai & Japanese DS-1-like G1P[8]
L3a (DS-1-like)
iii.
0.02
i.
Wa-like
(E1 Genotype)
DS-1-like (E2 Genotype)
RVA/Pigeon-tc/JPN/PO-13/1983/G18P[17]
0.1
Compressed NSP4 phylogram

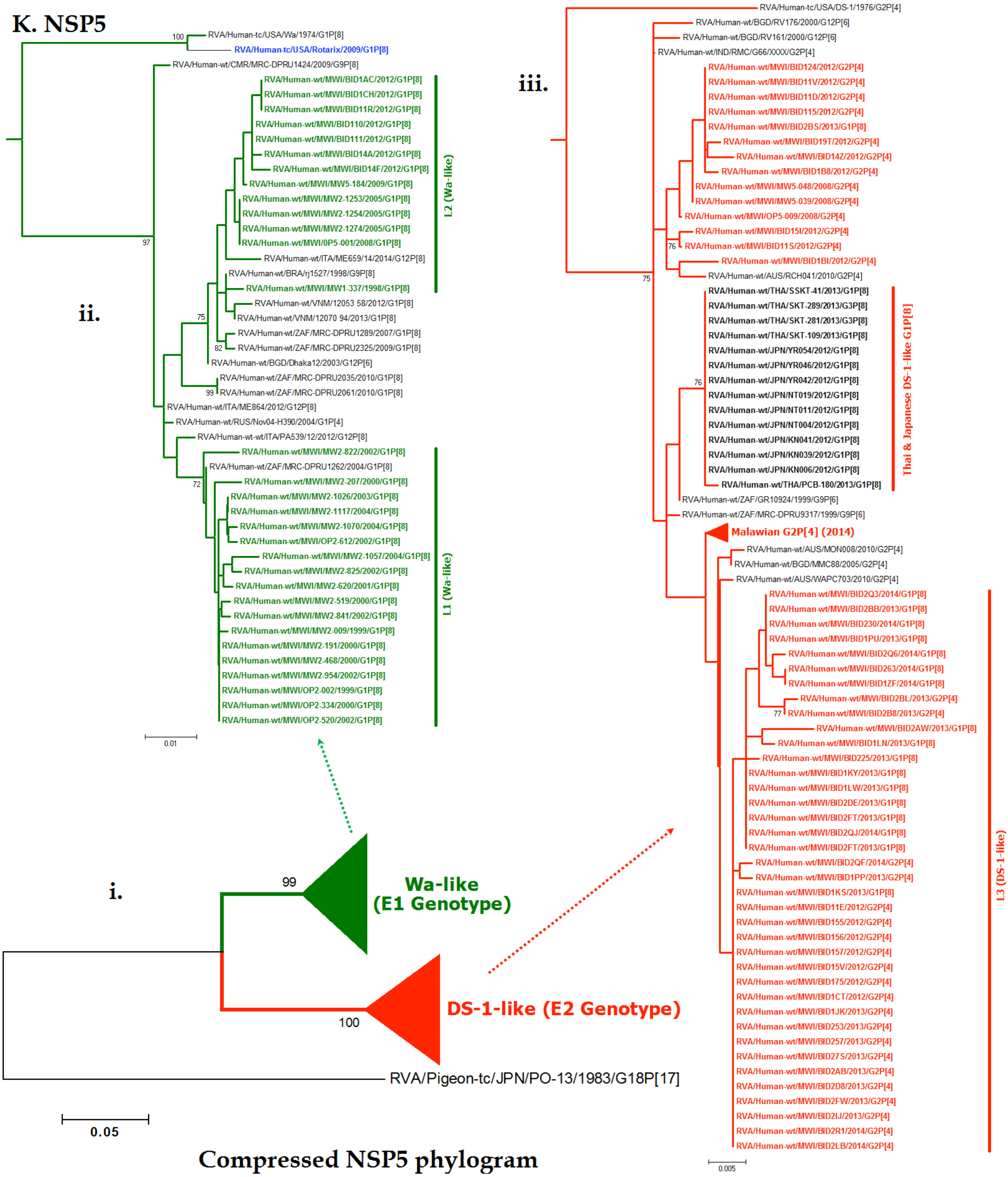

Compressed NSP5 phylogram

Fig. S2. Phylogenetic analysis of complete ORFs for individual 11 genome segments of Malawian G1P[8] strains compared to reference strains from elsewhere using Maximum-Likelihood method. Tree with the highest log likelihood are shown. The percentage of trees in which the associated taxa clustered together is shown next to the branches. Initial tree(s) for the heuristic search were obtained automatically by applying Neighbor-Joining and BioNJ algorithms to a matrix of pairwise distances estimated using the Maximum Composite Likelihood (MCL) approach, and then selecting the topology with superior log likelihood value. **(A – F)** Phylogenetic trees for genome segments 1 – 4, 6 and 9 that encodes rotavirus structural proteins (VP1 – VP4, VP6 and VP7), respectively. **(H–K)** Phylogenetic trees for genome segments 5, 8, 7, 10 and 11 that encodes rotavirus non-structural proteins (NSP1 – NSP5), respectively. All rotavirus strains are named according to the *Rotavirus Classification Working Group* (RCWG) guidelines where the nomenclature indicates the rotavirus group, host species, name of the country where the strain was originally isolated, the common name, year of isolation and the genotypes for genome segments encoding VP7 and VP4 (Matthijnssens et al., 2011), respectively. Taxa for study strains are highlighted in Green and red, and branches for Wa- and DS-1-like strains are shown in green and red, respectively. Taxa for vaccine strains is highlighted in blue whereas atypical G1P[8] strains from Asia are in bold. The horizontal branch lengths are proportional to the genetic distance calculated by the Maximum-likelihood method. The numbers adjacent to the node represents the bootstrap value of 1,000 replicates, and values less than 70% are not shown. The scale bar shows the genetic distance expressed as nucleotide substitution per rate of the nucleotide sequences.

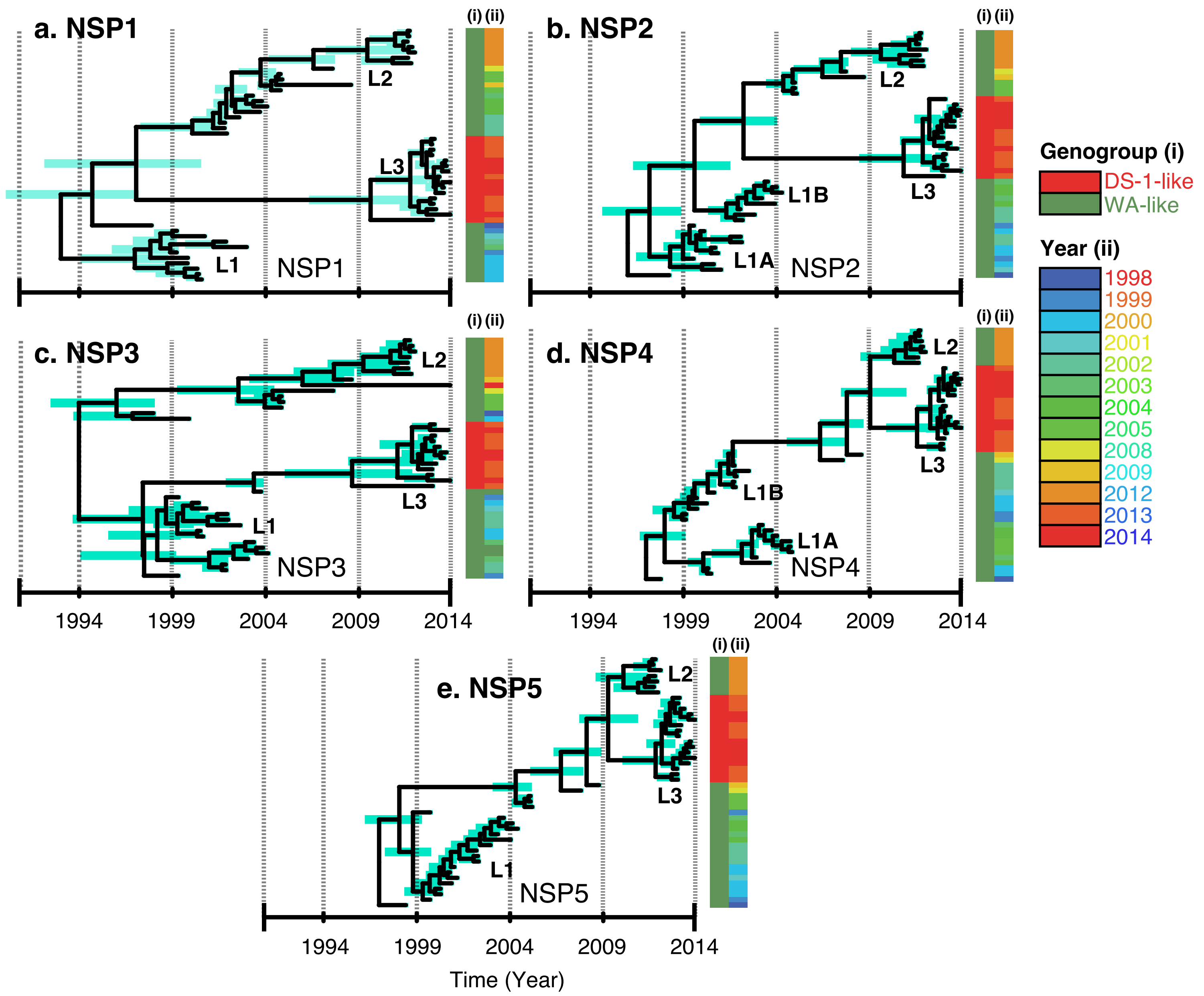

Fig. S3. Bayesian Maximum Clade Credibility (MCC) time tree based on complete nucleotide sequences illustrating lineage replacement within the genome segments encoding non-structural proteins of the G1P[8] strains that circulated in Malawi from 1998 – 2014. With the exception of NSP4 genes that had L1 sub-lineages, the rest had three main G1P[8] lineages. L1, L2 and L3 represents lineage 1, 2 and 3, respectively.

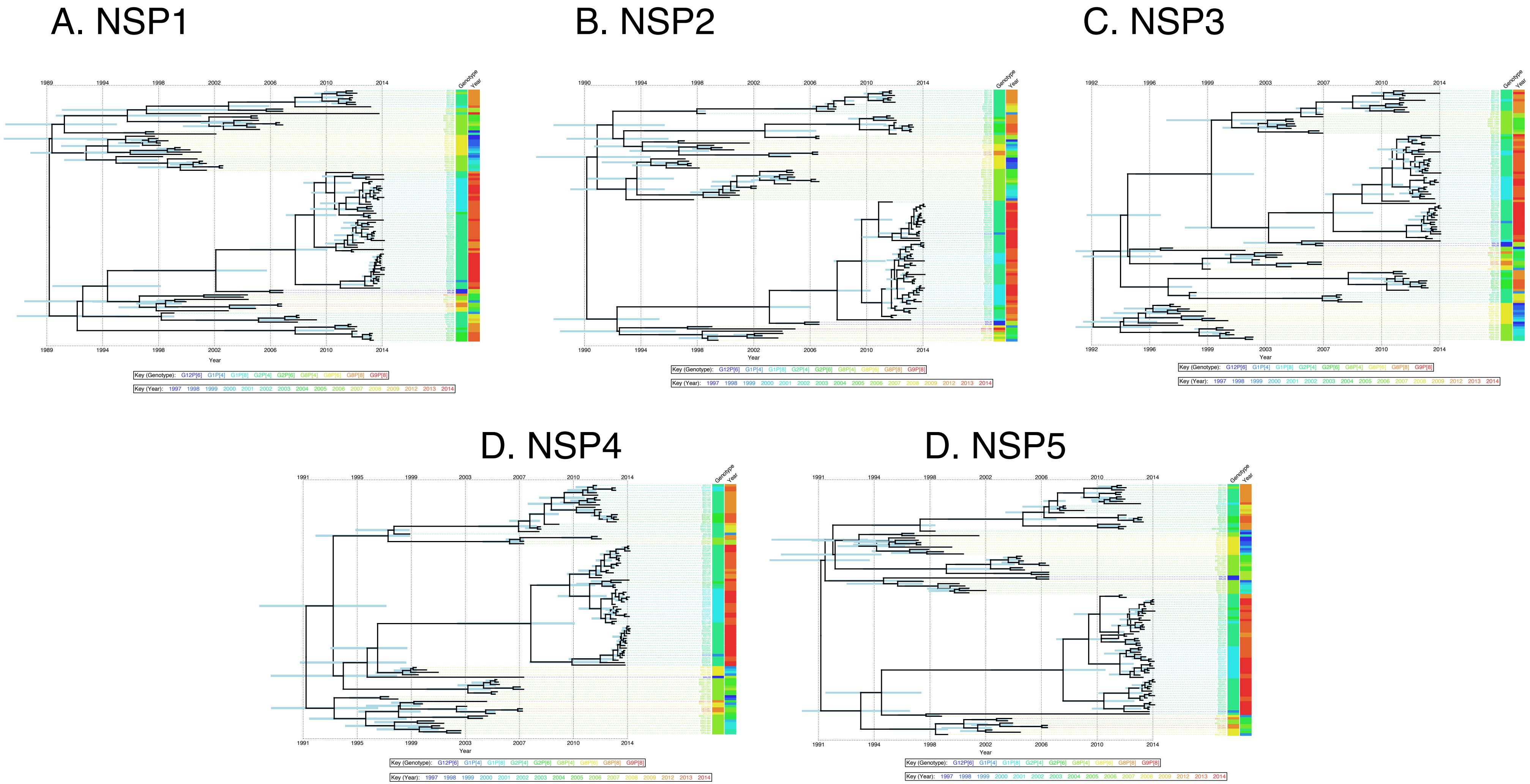

Fig. S4. Bayesian Maximum Clade Credibility (MCC) time tree based on complete nucleotide sequences of nonstructural proteins for G1P[8] strains from Malawi. Only DS-1-like genome segments for typical DS-1-like strains that were assigned G2P[4], G2P[6], G8P[4], G8P[6] and G12P[6] outer capsid genotypes from Malawi were included to calculate evolutionary dynamics for for genes encoding non-structural proteins (NSP1 – NSP5). The summary for their evolutionary rates and tMRCA are presented in Table 2.
